# Rovibrational dynamics of the quasistructural N_2_ dimer

**DOI:** 10.1038/s42004-025-01716-7

**Published:** 2025-11-07

**Authors:** Roland Tóbiás, Csaba Fábri, Marlene Bosquez, Monika Kodrycka, Konrad Patkowski, Attila G. Császár

**Affiliations:** 1 Institute of Chemistry, ELTE Eötvös Loránd University, Budapest, Hungary; 2 Department of Chemistry, University of Vermont, Burlington, VT USA; 3 Department of Theoretical Physics, University of Debrecen, Debrecen, Hungary; 4 ELTE Hevesy György PhD School of Chemistry, Budapest, Hungary; 5 Department of Chemistry and Biochemistry, Auburn University, Auburn, AL USA

**Keywords:** Computational chemistry, Chemical physics

## Abstract

Although the collision-induced absorption spectrum of the nitrogen gas is known in considerable detail, little has been learned experimentally about the structural, dynamical, and rovibrational characteristics of the nitrogen dimer itself. This study explores all these properties of this prototypical van der Waals (vdW) dimer and provides definitive quantum chemical results, mostly with attached conservative uncertainty estimates, particularly for the parent isotopologue, ^14^N_2_⋅^14^N_2_. The results obtained are based on three analytical representations of the dimer’s ground-state potential energy surface (PES), including two full-dimensional models of spectroscopic accuracy, constructed during the present study. The structural and focal-point analyses confirm that the global minimum of (N_2_)_2_ is planar and has a tilted, Z-shaped form, with an electronic dissociation energy of 109.3(26) cm^−1^. After considering zero-point vibrational effects variationally, the first dissociation limit of ^14^N_2_⋅^14^N_2_ is estimated to be 72.2(15) cm^−1^. The full- and reduced-dimensional variational nuclear-motion computations performed result in almost 6000 bound rovibrational states for ^14^N_2_⋅^14^N_2_, including over 100 purely vibrational modes. Effects arising from isotopic substitutions, as well as the shifts and splittings of the two quasi-bound N  ≡ N stretch fundamentals, are also examined. An in-depth analysis of the rovibrational eigenstates indicates that N_2_⋅N_2_ is a quasistructural molecular complex.

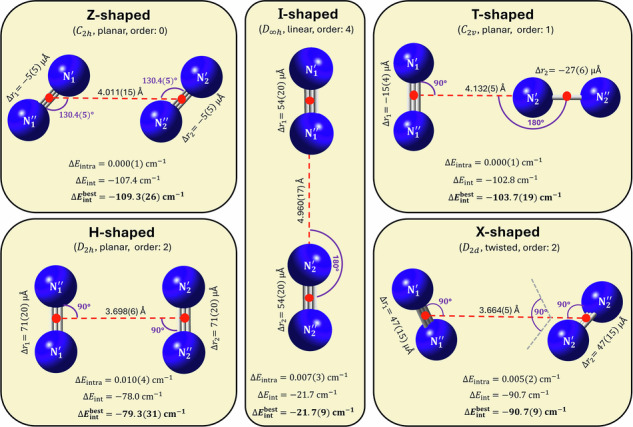

## Abstract (web summary)

Although the collision-induced absorption spectrum of molecular nitrogen has been studied in detail, rovibrational dynamics studies of the dimer remain scarce at best. Here, the authors report on the structure, rovibrational energy levels and nuclear dynamics of N_2_⋅N_2_, using newly developed 4D and 6D potential energy surfaces, resulting in a large number of bound rovibrational states and accurate shift and splitting values for the intramonomer (quasi-bound) stretch fundamentals.

## Introduction

Nitrogen gas is one of the principal atmospheric constituents not only on Earth, but also on exoplanets, moons, and even certain stars. As the parent isotopologue of the diatomic N_2_ molecule, ^14^N_2_, has no intrinsic dipole moment, its typical rovibrational spectral features are due to its nonzero quadrupole moment^1,2^. At a sufficiently high pressure, broad spectral features in the infrared region of the electromagnetic spectrum have been observed^3,4^ for molecules lacking permanent dipole moments, like H_2_, N_2_, O_2_, CH_4_, and their mixtures. At lower gas densities, these features are basically due to binary collisions governed by subtle non-covalent interactions (NCI)^5,6^, resulting in what are called collision-induced absorption (CIA) spectra^7^. For N_2_⋅N_2_, the collision-induced dipole arises from the polarization of one monomer by the quadrupolar field of the other one.

The CIA spectrum of the nitrogen gas was discovered in the laboratory in 1949^3^. This observation was followed by numerous experimental^8–24^ and theoretical/computational^25–28^ investigations. In particular, CIA spectra of the nitrogen gas have been measured both in the Earth’s atmosphere^20^ and in that of Saturn’s largest moon, Titan^11,19^. At low N_2_ concentrations, it is hard to record the CIA spectrum^3,18^, but it becomes much more visible in nitrogen-rich atmospheres, such as that of Earth. CIA even contributes to a small extent to the natural greenhouse effect^20^. It is worth mentioning that the CIA of the nitrogen gas had to be accounted for to explain that the atmosphere of early Mars was warm enough to support liquid water on its surface^29^.

Starting from its 2012 edition^30^, the canonical spectroscopic database HITRAN provides CIA-related parameters^22,30^. They are available for 20 binary systems in its latest version, HITRAN2020^31^, listed separately from the more usual line-by-line spectroscopic data. For the N_2_ dimer, there are more than 250000 CIA coefficients, collected from both experimental^13,16,17,21^ and theoretical/computational^23,27,28^ data sources, in diverse temperature ranges between 70 and 400 K. These data span the 0 – 650, 1850 – 3000, and 4300 – 5000 cm^−1^ spectral regions. The first wavenumber range contains the intermonomer vibrational bands of the N_2_ dimer, while the second and third intervals cover transitions involving the two intramonomer (N  ≡ N stretch) fundamentals and their first overtones, respectively. Some of these CIA coefficients may have limited accuracy, due to (a) experimental uncertainties, often caused by ill-resolved overlapping lines and/or contaminant species, and (b) computational artifacts, arising from the use of inaccurate potential energy (PES) and property surfaces (often in combination with deficiencies of the dynamical models applied).

The first step toward the accurate first-principles computation of CIA spectra is the determination of an accurate potential energy surface (PES). About this a lot of experience has been gained over the past few decades, especially for binary van der Waals complexes; see, for example, refs. ^32–44^. To build reliable analytical PESs for NCI complexes like N_2_⋅N_2_, highly flexible model functions must be selected, trained on NCI energies at a large number of grid points. For the accurate computation of these rather small NCI energies, not only electron-correlation effects have to be taken into account, but the basis-set incompleteness and superposition errors should also be minimized. All this has usually been achieved *via* the “gold standard” CCSD(T) (that is, the coupled cluster singles, doubles, and iterative triples) method^45^, combined with extrapolation^46,47^ and counterpoise-correction^48^ schemes. To increase accuracy, consideration of so-called “small corrections”^49,50^ may also become necessary. Convergence of the individual correlation-energy increments to relative energies can be traced with the help of the focal-point analysis (FPA) scheme^49,51^, yielding the ultimate first-principles estimates, with definitive uncertainties^52^, for the NCI energies at particular configurations or over the entire PES^53^. If coupled-cluster computations are unaffordable for a complex, symmetry-adapted perturbation theory (SAPT) protocols^54–58^ provide excellent alternatives.

Over the past few decades, several first-principles and empirical PESs have been developed for the N_2_ dimer^59–73^, though most of them within the rigid-monomer approximation (*i.e*., keeping the two N≡N bond lengths fixed, resulting in four-dimensional (4D) dynamical models). Exceptions are the two full-dimensional (6D) PESs of the Truhlar group^74,75^, designed for the investigation of high-energy rovibrational energy transfer and collision-induced monomer dissociation in the N_2_⋅N_2_ system. These PESs, unfortunately, have only chemical (~ 350 cm^−1^) accuracy. Among the 4D surfaces, Hellmann’s scaled benchmark PES^72^ has the highest accuracy, approaching the full configuration interaction (FCI) and complete basis set (CBS) limits^33,53^ and involving small corrections^49^ at vibrationally averaged monomer bond lengths. This 4D PES leaves no doubt that the global minimum of the N_2_ dimer is a planar tilted structure of *C*_2*h*_ point-group symmetry, in contrast to PESs exhibiting T-shaped^62,66,71^ or nonplanar “twisted”^59,60,63^ global minima. Such discrepancies can be ascribed to the lack of accurate first-principles data points and the use of over-simplified functional forms^76^.

Despite the extensive literature available on other binary complexes, see, *e.g*., refs. ^32,37,38,40,41,43,44^, to the best of our knowledge, there are only three articles^66,77,78^ reporting bound-state rovibrational computations on N_2_⋅N_2_. In a first, ground-breaking study, Tennyson and van der Avoird^77^ employed a rigid-monomer Hamiltonian and a simple 4D PES^59^, with a twisted global minimum of *D*_2*d*_ point-group symmetry (this PES was fitted to SAPT-like computations involving Hartree–Fock monomer wavefunctions and small Gaussian basis sets). This analysis produced a large number of bound states for *J* ≤ 2 (specifically, 92 for *J* = 0), where *J* denotes the overall rotational quantum number. Next, Brocks and van der Avoird^78^ simulated the far- and mid-infrared spectra of ^14^N_2_⋅^14^N_2_, using the formalism of ref. ^77^, up to *J* = 7. A similar protocol was followed by Aquilanti et al., but with a fully empirical PES parametrized for scattering experiments, whose global minimum has a T-shaped form^66^. They computed bound rovibrational states up to *J* = 6. Unfortunately, all of these otherwise sophisticated studies relied on qualitatively incorrect PESs, as the global minimum is neither twisted nor T-shaped^72,76^.

In this work, the structure, the rovibrational energy levels, and the nuclear dynamics of N_2_⋅N_2_ are reconsidered, using exact kinetic-energy operators and highly accurate PESs. The potentials used include Hellmann’s 4D PES^72^ and two newly created 6D PESs, with the more accurate one having spectroscopic (1 cm^−1^) accuracy. The present investigation delivers an exhaustive list of bound rovibrational states for the ^14^N_2_⋅^14^N_2_ dimer up to *J* = 10, with state-by-state uncertainty estimates and correct symmetry labels corresponding to the irreducible representations of the *G*_16_ molecular symmetry group^79^. For the first time, accurate shifts and splittings are deduced for the intramonomer stretch fundamentals, representing quasi-bound (resonance) states. For the lowest doubly degenerate vibrational state of ^14^N_2_⋅^14^N_2_, isotope effects are also considered. Based on our extensive computational results, it can be firmly established that N_2_⋅N_2_ behaves as a quasistructural^80^ complex, whose highly unusual and interesting rovibrational states fail interpretation attempts based on the simplest rigid rotor and harmonic oscillator models.

## Results and discussion

Perhaps the most significant finding of this article is that it provides clear evidence for the quasistructural nature^80^ of the nitrogen dimer, a prototype of weakly-bound vdW dimers. This conclusion is based on cutting-edge electronic-structure and quasi-)variational nuclear-motion computations^81^, employing exact kinetic-energy operators both in reduced and full dimensions. Following some introductory remarks, our discussion focuses only on the analysis of the numerical results, while the important technical details about the computational methodology employed are contained in Section “Methods” and in Supplementary Notes 1–4. Apart from Section “Isotope effects”, our rovibrational and dynamical results concern the parent N_2_⋅N_2_ isotopologue, ^14^N_2_⋅^14^N_2_.

**Fig. 1:**
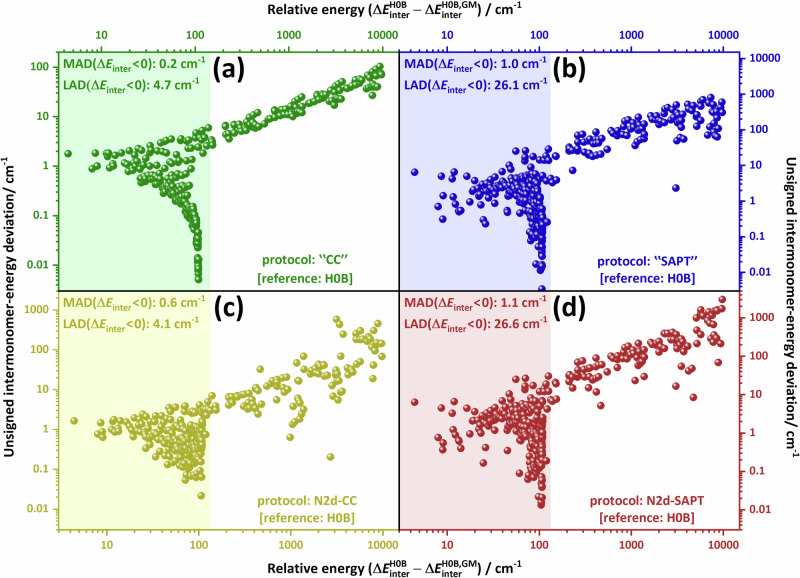
Comparison of the intermonomer energies of the N_2_ dimer using various levels of electronic structure theory. The panels depict the deviations of the direct electronic-structure results (**a**, **b**) and their fitted counterparts (**c**, **d**) from the H0B reference values. For the “C”, “SAPT”, N2d-CC, N2d-SAPT, and H0B protocols, see Section “Methods”. The reference grid points and their benchmark (H0B) intermonomer energies are taken from ref. ^72^, whereas the other (non-H0B) data were obtained during the present study. The relative energies are taken with respect to the intermonomer energy at the global minimum of the H0B energy scheme, with $\Delta {E}_{{{\rm{inter}}}}^{{{\rm{H0B,GM}}}}\approx -109.21$ cm^−1^ ^72^. The colored boxes on the left-hand side of each panel highlight points whose intermonomer energies are below the dimer’s approximate dissociation limit, $-\Delta {E}_{{{\rm{inter}}}}^{{{\rm{H0B,GM}}}}$. In these boxes, the median absolute deviations (MAD) and the largest absolute deviations (LAD) pertaining to dimer geometries with negative intermonomer energies are also given.

**Fig. 2:**
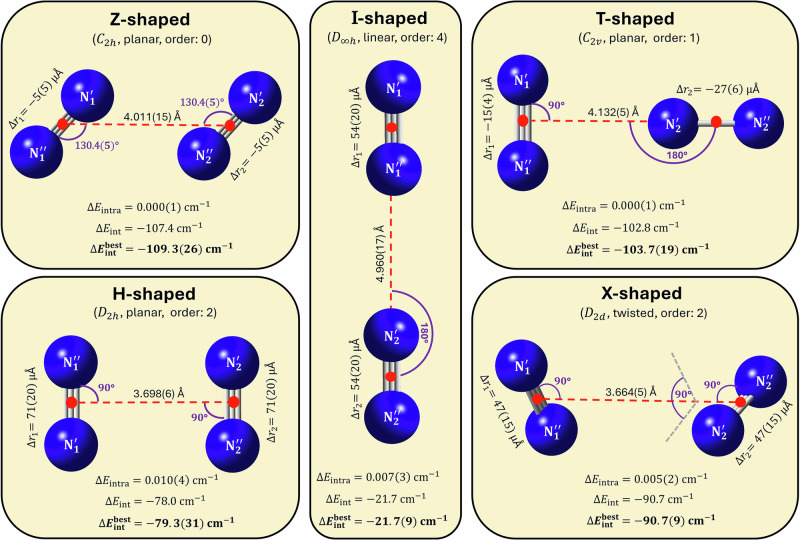
Five salient stationary points on three analytical potential energy surfaces (PES) of N_2_⋅N_2_. For parameter values affected by computational error, the last-digit (two-sigma) uncertainties are given in parentheses. The boldfaced $\Delta {E}_{{{\rm{int}}}}^{{{\rm{best}}}}$ values represent the best estimates of this study. All the other parameter values correspond to the N2d-CC PES [see also Section “Potential energy surfaces and stationary points” and Supplementary Note 1]. The stationary-point order, *i.e*., the number of negative Hessian eigenvalues, is given for each shape next to its point-group symmetry. As customary, the intermonomer coordinates (see Section “Internal coordinates and nuclear-motion models”) are indicated with (dashed) lines and arcs. Instead of the *r*_1_ and *r*_2_ bond lengths, the Δ*r*_1_ and Δ*r*_2_ relative values are displayed, respectively (these relative distances are referenced to the equilibrium bond length of the isolated N_2_ unit; see Section “Internal coordinates and nuclear-motion models”). The intramonomer energies, Δ*E*_intra_, are negligibly small for all five geometries (see Section “PES construction”). The uncertainty estimates are based on the deviations between the N2d-SAPT/H0B and N2d-CC results.

First, results of rigorous tests performed to determine the level of electronic-structure theory needed to obtain a highly accurate, spectroscopically meaningful, full-dimensional PES for N_2_⋅N_2_ are discussed (see Fig. 1). Second, five salient stationary points on the PES of N_2_⋅N_2_, Z, T, X, H, and I (see Fig. 2), are investigated. Third, results from our benchmark-quality first-principles rovibrational computations, yielding a large number of bound and a few resonance (quasi-bound)^82^ states are summarized. Fourth, the peculiar characteristics of these (ro)vibrational quantum states are explored *via* probability-density analyses, offering an interpretation of the computational results.

### Potential energy surfaces and stationary points

To decide about the level of electronic-structure theory that should be used to develop an analytical PES for N_2_⋅N_2_ with spectroscopic (≈ 1 cm^−1^) accuracy, the computational results of Hellmann^72^, called here H0B and produced at a high, composite level of electronic-structure theory, have been taken as reference values. Since our aim has been to supervise the construction of the PES with the autoPES program system^35,39^, used successfully by us during a similar project^83^, the present analysis focuses on levels of electronic-structure theory directly supported by this package through its interfaces. Specifically, two levels are considered, referred to as “SAPT” and “CC” (always with double quotes) throughout this paper (for technical details about the H0B, “SAPT”, and “CC” levels, see Section “Electronic-structure computations”).

Figure 1 illustrates the unsigned deviations of our intermonomer energies from their H0B counterparts at grid points selected, up to 10,000 cm^−1^, from the list of Hellmann^72^. In Fig. 1(a), a remarkably good agreement is seen between the results of the coupled-cluster-based “CC” and the H0B protocols, translating to sub-cm^−1^ agreement for the majority of the structures with dominant vdW attractions. As apparent from Fig. 1(b), the “SAPT” energies also agree well with Hellmann’s benchmark values, though the differences are mostly larger here than in the “CC” case. This encouraged us to create two 6D surfaces, named N2d-CC and N2d-SAPT, fitted to “CC” and “SAPT” energies, respectively, whose parametrization did not benefit from Hellmann’s grid points. The two PESs nicely reproduce the H0B energies, to the same extent as the direct “CC”/"SAPT” computations [see Fig. 1(c)/(d)]. These two PESs (see Section “PES construction” how they were created), along with Hellmann’s original (H0B-based) PES designated here as N2d-H0B, have been applied in our rovibrational and dynamical analyses.

To explore the stationary points (SP) of the N2d-H0B, N2d-CC, and N2d-SAPT PESs, extensive global searches have been made, revealing altogether five salient SPs with relatively high point-group symmetries (see Fig. 2). These five nuclear arrangements could be identified as SPs on all three PESs and their orders are consistent across the different PESs. To name these SPs, the convention applied in ref. ^74^ has been adopted (see Fig. 2). Our results show that (a) the tilted, Z-shaped form is the only minimum on these three PESs, and (b) the intramonomer effects are minuscule, at most 0.05 cm^−1^ for the relative interaction energies, for the five SPs considered (see Fig. 2 and Supplementary Table 2 for the structural and interaction parameters of these SPs).

As a next step, detailed FPA analyses^49,51^ were performed for three of the five SPs to further validate the accuracy of the N2d-CC/SAPT PESs (see Supplementary Note 1). The resulting interaction energies, alongside their estimated uncertainties in parentheses, are collected in Supplementary Table 2 (note that part of the electronic-structure results concerning the global minimum was taken from ref. ^36^). The various interaction-energy determinations agree nicely: in fact, almost all of the deviations fall within or are at least reasonably close to the ultimate FPA uncertainties. As to the correlation-energy increments given in Supplementary Table 1 for the three SPs studied *via* FPA, each post-CCSD(T) term is below  ± 6 cm^−1^, and they have rather similar values, mostly with the same signs. Thus, these contributions cancel each other to a large extent in the relative interaction energies, a typical effect for non-covalent complexes^84^.

### First-principles rovibrational results

Making use of the three PESs discussed in Sec. 2.1, variational nuclear-motion computations, always with exact kinetic-energy operators, have been carried out, exploiting the permutation-inversion symmetry of the dimer (see Section “Symmetry-adapted variational nuclear-motion computations”). This subsection gives a description of the computational results obtained, including accurate rovibrational energies of the ^14^N_2_⋅^14^N_2_ complex both below and above the first dissociation limit, corresponding to bound and resonance states, respectively. The results reported take advantage of four dynamical models of different dimensionality, that is 6D, 4D, 4D0, and 2D, introduced for the N_2_ dimer in Section “Internal coordinates and nuclear-motion models”. The discussion itself is divided into four parts, focusing on the zero-point vibrational effect, the bound and then the resonance states, and on isotope effects.

#### Zero-point vibrational effects

Employing the FPA protocol^49,51^, the electronic interaction energy, Δ*E*_int_, is estimated to be  −109.3 ± 2.6 cm^−1^ for the global minimum of N_2_⋅N_2_. Compared to this value, the zero-point vibrational energy (ZPVE) correction, $\delta {E}_{{{\rm{int}}}}^{{{\rm{ZPVE}}}}$, is substantial (see Table 1); thus, knowing its value accurately is essential to characterize the thermodynamic stability of the N_2_ dimer at absolute zero temperature. In particular, the sum of these two terms is the interaction (Gibbs) free energy at 0 K, $\Delta {G}_{{{\rm{int}}}}^{{{\rm{0}}}}$, whose absolute value equals the first dissociation limit of (N_2_)_2_ (this quantity is also needed to find the highest-energy bound state in the dimer’s energy spectrum for each *J* value).

**Table 1:** Electronic and zero-point vibrational effects in the Z-shaped global minimum of the N_2_ dimer^〈*a*〉^

Potential^〈*b*〉^	$\Delta {{E}_{{{\rm{int}}}}}^{\langle c\rangle }$	$\delta {{E}_{{{\rm{int}}}}^{{{\rm{ZPVE}}}}}^{\langle d\rangle }$			$\Delta {{G}_{{{\rm{int}}}}^{{{\rm{0}}}}}^{\langle e\rangle }$			${{\rm{ZPVE}}}{({{{\rm{N}}}}_{2}\cdot{{{\rm{N}}}}_{2})}^{\langle f\rangle }$	
		4D	4D0	6D	4D	4D0	6D	2D + 4D	6D
N2d-H0B	–109.2		35.5			–73.7			
N2d-CC	–107.4	35.4	35.2	35.2	–72.0	–72.2	–72.2	2387.0	2386.7
N2d-SAPT	–106.3	33.9	33.6	33.6	–72.4	–72.7	–72.7	2385.4	2385.1
Best^〈*g*〉^	–**109.3(26)**			**35.2(3)**			–**72.2(15)**		**2386.7(16)**

^〈*a*〉^All numerical values are given in cm^−1^.

^〈*b*〉^The analytical potential energy surfaces are defined in Section “Potential energy surfaces and stationary points”.

^〈*c*〉^Estimates for the interaction energy of the Z-shaped global minimum from Supplementary Table 2.

^〈*d*〉^First-principles zero-point vibrational energy (ZPVE) corrections determined with different potentials and dynamical models (see also Section “Internal coordinates and nuclear-motion models”). $\delta {E}_{{{\rm{int}}}}^{{{\rm{ZPVE}}}}$ refers to the change due to the intermolecular vibrational modes.

^〈*e*〉^Variational estimates for the zero-point-corrected interaction energy (i.e., the interaction free energy at 0 K).

^〈*f*〉^Variational ZPVE values for the Z-shaped global minimum. 2D + 4D means the sum of the ZPVEs corresponding to the 2D and 4D dynamical models.

^〈*g*〉^Best predictions obtained for the four quantities are highlighted in boldface and their expanded (two-sigma) uncertainties are in parentheses.

Table 1 lists various predictions for three quantities, Δ*E*_int_, $\delta {E}_{{{\rm{int}}}}^{{{\rm{ZPVE}}}}$, and $\Delta {G}_{{{\rm{int}}}}^{{{\rm{0}}}}$, extracted from our electronic structure and variational nuclear-motion computations. Apparently, the variational $\delta {E}_{{{\rm{int}}}}^{{{\rm{ZPVE}}}}$ and $\Delta {G}_{{{\rm{int}}}}^{{{\rm{0}}}}$ values barely change across the different dynamical models and PESs: their maximum unsigned deviations are as low as 1.1 and 1.5 cm^−1^, respectively (based on the deviations observed, uncertainty estimates have been adopted for the best predictions of all these quantities). A harmonic force field, computed at the frozen-core CCSD(T) level using an augmented quadruple-zeta basis set^85^, provides $\delta {E}_{{{\rm{int}}}}^{{{\rm{ZPVE}}}}$ = 49.2 cm^−1^, which differs significantly, by as much as 14 cm^−1^, from our accurate variational estimate, 35.2(3) cm^−1^. Interestingly, previous $\Delta {G}_{{{\rm{int}}}}^{{{\rm{0}}}}$ predictions,  −79.8^66^ and  −75.0^78^ cm^−1^, are reasonably close to our best estimate,  − 72.2(15) cm^−1^, although they are based, respectively, on PESs with (incorrect) T- and X-shaped global minima (the small deviations observed are most likely due to the extremely similar interaction energies characterizing these qualitatively different structures).

#### Bound states

**Fig. 3:**
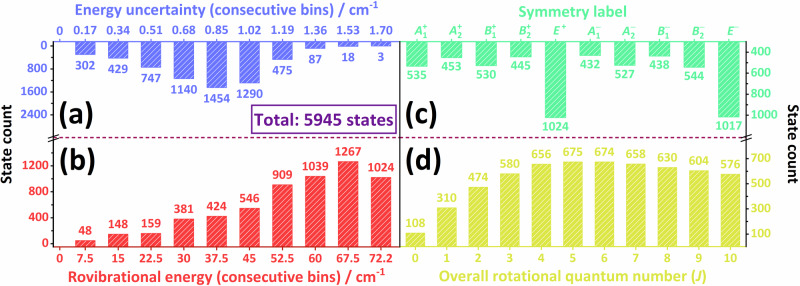
Statistical characteristics of the bound rovibrational states obtained during this study. In (**a**, **b**), the range represented by a bin is given by the actual and the previous axis ticks (e.g., the blue bin at 0.17 cm^−1^ contains states with an uncertainty of 0.00–0.17 cm^−1^). In contrast, the horizontal axes of (**c**, **d**) have discrete (integer) values. The state counts, *i.e*., the bin sizes, are shown on a unified bi-directed vertical axis for both pairs of distributions.

Accurate rovibrational energies have been computed for nearly 6000 bound states of ^14^N_2_⋅^14^N_2_, taking advantage of our improved methodology described in Section “Symmetry-adapted variational nuclear-motion computations”. The list of the computed energy levels, including all rovibrational states with *J* ≤ 10 below the first dissociation limit, is given in an external repository^86^ through an Excel file. Readers interested in individual characteristics of these bound states, such as the “raw” energy values produced *via* various dynamical models and PESs, are invited to study that file. In what follows, our discussion is focusing on some overall statistical measures, illustrated in Fig. 3.

On the left two panels of Fig. 3 the state distributions represent two continuous measures, namely the rovibrational energies [panel (b)] and their uncertainties [panel (a)]. As expected, the overwhelming majority of the bound states, more than 80 % of them, fall above half of the dissociation energy. Nevertheless, there are also dozens of states in the lowest energy bin, 0–7.5 cm^−1^, reflecting the weakly-bound nature and the relative heaviness of N_2_⋅N_2_. Most of the uncertainty, defined in Eqs. (4) and (5) and stretching over the 0–1.7 cm^−1^ range, comes from intermonomer effects, while the error contributions due to intramonomer motions remain below 0.15 cm^−1^.

The two right-hand-side panels of Fig. 3 are based on two discrete variables, namely the *J* values [panel (d)] and the symmetry blocks [*i.e*., irreducible representations (irreps) of the *G*_16_ group, panel (c)]. The distribution along the *J* values has a maximum at *J* = 5: from that point on, the (2*J* + 1)-fold rotational degeneracy ceases to be the dominant factor over the energy cutoff represented by the dissociation energy. It is of interest to observe in Fig. 3(c) that the blocks *E*^+^ and *E*^−^, even though their two-fold degenerate states are counted only once, are about twice as large as the other blocks. The small energy of the lowest *E*^+^ state may explain this (see also Section “Isotope effects”), as it can form several combination bands with other one-dimensional irreps.

#### Resonance states

Among the large number of resonance states^82^ one may compute for ^14^N_2_⋅^14^N_2_, attention is focused here only on the two long-lived intramonomer stretch fundamentals. The symmetric (sSTRE) and the antisymmetric (aSTRE) stretch states transform according to the ${A}_{1}^{+}$ and ${B}_{2}^{+}$ irreps of the *G*_16_ molecular symmetry group, respectively. Since the sSTRE and aSTRE states lie at least 30 times higher than the dissociation limit, their computation was greatly accelerated by the exploitation of the block-diagonal structure of the Hamiltonian; in particular, our computations were restricted to the ${A}_{1}^{+}$ and ${B}_{2}^{+}$ blocks in this energy domain, a considerable gain in efficiency. The relatively small basis set sufficient to achieve converged sSTRE/aSTRE energies also helped (see Section “Methods”).

Table 2 includes multiple determinations for the shifts and splittings of the sSTRE and aSTRE fundamentals, all relative to the energy of the fundamental of the N_2_ monomer. The best results, corresponding to the full-dimensional N2d-CC PES, are given in the last row of Table 2 (typeset in boldface). Clearly, there are pronounced changes in these shifts/splittings across the different PESs and their intermediate versions; thus, estimating the uncertainties of these computed quantities is exceedingly difficult (the value of  ±  0.1 cm^−1^, stated in Table 2, is a conservative estimate). As the even larger errors of the 2D predictions suggest, the intra- and intermonomer couplings result in non-negligible contributions to these quantities.

**Table 2:** Computed shifts and splittings in the two intramonomer stretch fundamentals of ^14^N_2_⋅^14^N_2_^〈*a*〉^

Estimate^〈*e*〉^	Shift(sSTRE)^〈*b*〉^[${A}_{1}^{+}$]		Shift(aSTRE)^〈*c*〉^[${B}_{2}^{+}$]		Splitting^〈*d*〉^	
	2D	6D	2D	6D	2D	6D
Range(N2d-SAPT)	[0.10, 0.32]	[–0.36, –0.18]	[–0.29, –0.06]	[–0.43, –0.30]	[–0.41,–0.27]	[–0.12, –0.05]
Best(N2d-SAPT)	0.13	–0.33	–0.17	–0.38	–0.30	–0.05
Range(N2d-CC)	[0.10, 0.31]	[–0.21, –0.15]	[–0.25, –0.09]	[–0.35, –0.28]	[–0.40, –0.34]	[–0.17, –0.10]
Best(N2d-CC)	0.26	–**0.19(10)**	–0.09	–**0.28(10)**	–0.35	–**0.10(10)**

^〈*a*〉^All numerical data are in cm^−1^. The full-dimensional ‘best’ cases are attached with expanded (two-sigma) uncertainty estimates.

^〈*b*/*c*〉^Shift of the symmetric/antisymmetric stretch (sSTRE/aSTRE) fundamental, relative to its counterpart in the free N_2_ molecule, 2329.912 cm^−1^ ^1^. The symmetry labels of the sSTRE/aSTRE states, within the *G*_16_ molecular symmetry group, are given in brackets. For the 2D/6D model, see Section “Internal coordinates and nuclear-motion models”.

^〈*d*〉^Energy difference defined as aSTRE—sSTRE.

^〈*e*〉^Estimates using the N2d-CC and N2d-SAPT potential energy surfaces (PES). The best values, typeset in boldface, are derived *via* the final versions of the two PESs, while the ranges are deduced from five intermediate PES versions produced during the PES-refinement process.

Although the final computed shifts and splittings are small, below 1 cm^−1^, they are significant given the accuracy and resolution of today’s spectroscopic measurements. Accordingly, it seems feasible to generate experimental information about the energy ordering of the free stretch of N_2_ and the sSTRE and aSTRE fundamentals of ^14^N_2_⋅^14^N_2_, however small the differences are.

#### Isotope effects

Table 3 shows the effects of isotopic substitution for the lowest two excited vibrational states of the six possible ^14^N and ^15^N isotopologues, with symmetry labels provided for each case. The double degeneracy, characterizing these two vibrational modes in the ^14^N_2_⋅^14^N_2_ dimer, is lifted for the mixed isotopologues, where both ^14^N and ^15^N are present, inducing small shifts and splittings. The largest shift is, of course, for the fully symmetric ^15^N_2_⋅^15^N_2_ isotopologue, for which there is again no splitting. These quantities turned out to be insensitive to the details of the PES used for their computation, leading to estimated uncertainties on the order of 10^−3^ cm^−1^ (see Table 3).

**Table 3:** Isotope effects in the first two excited vibrational states of the N_2_ dimer^〈*a*〉^

Species^〈*b*〉^	Group^〈*c*〉^	${{{{\rm{vib}}}}_{1}}^{\langle d\rangle }$			${{{{\rm{vib}}}}_{2}}^{\langle e\rangle }$			Splitting^〈*i*〉^
		Label^〈*f*〉^	Shift^〈*g*〉^	Dev.^〈*h*〉^	Label^〈*f*〉^	Shift^〈*g*〉^	Dev.^〈*h*〉^	
^14^N_2_ ⋅ ^14^N_2_	*G*_16_	*E*^+^	0.0	0.0	*E*^+^	0.0	0.0	0.0
^14^N_2_ ⋅ ^14^N^15^N	*G*_4_	*A*^+^	–0.111(1)	0.001	*B*^+^	–0.060(2)	0.001	0.051(1)
^14^N_2_ ⋅ ^15^N_2_	*G*_8_	${B}_{2}^{{\prime\prime} }$	–0.226(3)	–0.001	${B}_{2}^{{\prime} }$	–0.123(1)	–0.001	0.103(4)
^14^N^15^N ⋅ ^14^N^15^N	*G*_4_	*B*^+^	–0.171(2)	0.002	*A*^+^	–0.170(3)	0.003	0.001(1)
^14^N^15^N ⋅ ^15^N_2_	*G*_4_	*B*^+^	–0.285(1)	0.001	*A*^+^	–0.233(2)	0.001	0.052(3)
^15^N_2_ ⋅ ^15^N_2_	*G*_16_	*E*^+^	–0.346(2)	0.001	*E*^+^	–0.346(2)	0.001	0.0

^〈*a*〉^All numerical data are given in cm^−1^, with the (two-sigma) uncertainties of their last few digits in parentheses. For the calculation of these uncertainties, Eq. (4) was applied, but replacing the absolute energies with shifts/splittings.

^〈*b*〉^Isotopologue of the N_2_⋅N_2_ complex, composed of ^14^N and/or ^15^N isotopes with spin-1 and spin-1/2 nuclei, respectively.

^〈*c*〉^Molecular symmetry group of a particular species.

^〈*d*/*e*〉^First/second excited vibrational state of a given isotopologue.

^〈*f*〉^Symmetry label associated with a specific state of this table.

^〈*g*〉^Shifts of the first two excited vibrational energies with respect to their degenerate sibling in the ^14^N_2_⋅^14^N_2_ species, 3.57(3) cm^−1^.

^〈*h*〉^Deviation of a shift predicted *via* Eq. (1) from its variational counterpart. The monomer masses behind Eq. (1) rely on ^14^N and ^15^N masses defined in Section “Methods”.

^〈*i*〉^Energy splitting between vib_1_ and vib_2_, which is equivalent to shift(vib_1_) – shift(vib_2_).

As expected, the shifts of Table 3 are always negative, and their absolute values increase with the number of ^15^N atoms in the dimer. Assuming $\Delta {{{\mathcal{M}}}}_{1}\le \Delta {{{\mathcal{M}}}}_{2}$, where $\Delta {{{\mathcal{M}}}}_{i}$ is the change in the mass of the *i*th monomer compared to that of the ^14^N_2_ isotopologue, the shifts of the vibrational modes vib_1_ and vib_2_ closely follow a simple model,

1 $${{\rm{shift}}}({{{\rm{vib}}}}_{i})\approx {a}_{1}\,\Delta {{{\mathcal{M}}}}_{i}+{a}_{2}\,\Delta {{{\mathcal{M}}}}_{3-i},$$

where *a*_1_ = −0.060 8(9) cm^−1^ u^−1^ and *a*_2_ = −0.112 5(9) cm^−1^ u^−1^ are two fitted parameters, which perfectly coincide with the two shifts of the ^14^N_2_⋅^14^N^15^N complex [see Supplementary Note 4 for the derivation of Eq. (1)]. These shifts and splittings are straightforwardly measurable; thus, their accurate estimates given in Table 3 should become useful guides during the interpretation of the related spectroscopic experiments.

### Manifestation of quasistructurality of the N_2_ ⋅ N_2_ complex

In ref. ^80^, it was proposed that a molecular system should be called quasistructural if it satisfies all of the following five criteria: (i) “the notion of a static equilibrium structure, corresponding to a minimum on the potential energy surface of the molecule, loses its strict meaning”, (ii) “internal nuclear motions [...] become dominant, resulting in an effective molecular structure often even qualitatively different from the equilibrium one”, (iii) “separation of the internal nuclear motions breaks down, rotational and vibrational degrees of freedom cannot be separated from each other when interpreting even the lowest rovibrational eigenstates of the molecule, often resulting in effective rotational constants drastically different from the equilibrium ones even for the ground vibrational eigenstate”, (iv) “classification of the rovibrational states requires the use of permutation-inversion symmetry”, and (v) “some of the rovibrational eigenenergies assigned to a vibrational parent state exhibit unconventional [...] rotational contributions”. In the upcoming analysis, our aim is to study how closely these criteria are met for N_2_⋅N_2_.

It is important to clarify the relation of quasistructural molecules^80^ to floppy and fluxional/fluctional^87^ systems. In floppy molecules, there are one or more large-amplitude internal motions, but their rotational energy-level structure may be fitted well by a rigid or a semirigid effective Hamiltonian. As noted by Bunker and Jensen^79^ about ethane, “except in ultrahigh resolution spectroscopic studies ethane can be considered to be a rigid molecule.” Fluxional systems exhibit rapid, degenerate rearrangements among physically equivalent structures. Although quasistructural molecules necessarily fall under the broader categories of floppy or fluxional systems, the converse is not true. Quasistructurality denotes a more specific structural and dynamical behavior, one accompanied by a qualitative breakdown of standard spectroscopic paradigms. In particular, the commonly assumed separation of rotational and vibrational motion is already not valid even for the lowest-energy states (see below).

#### Extremely flat interaction PES

**Fig. 4:**
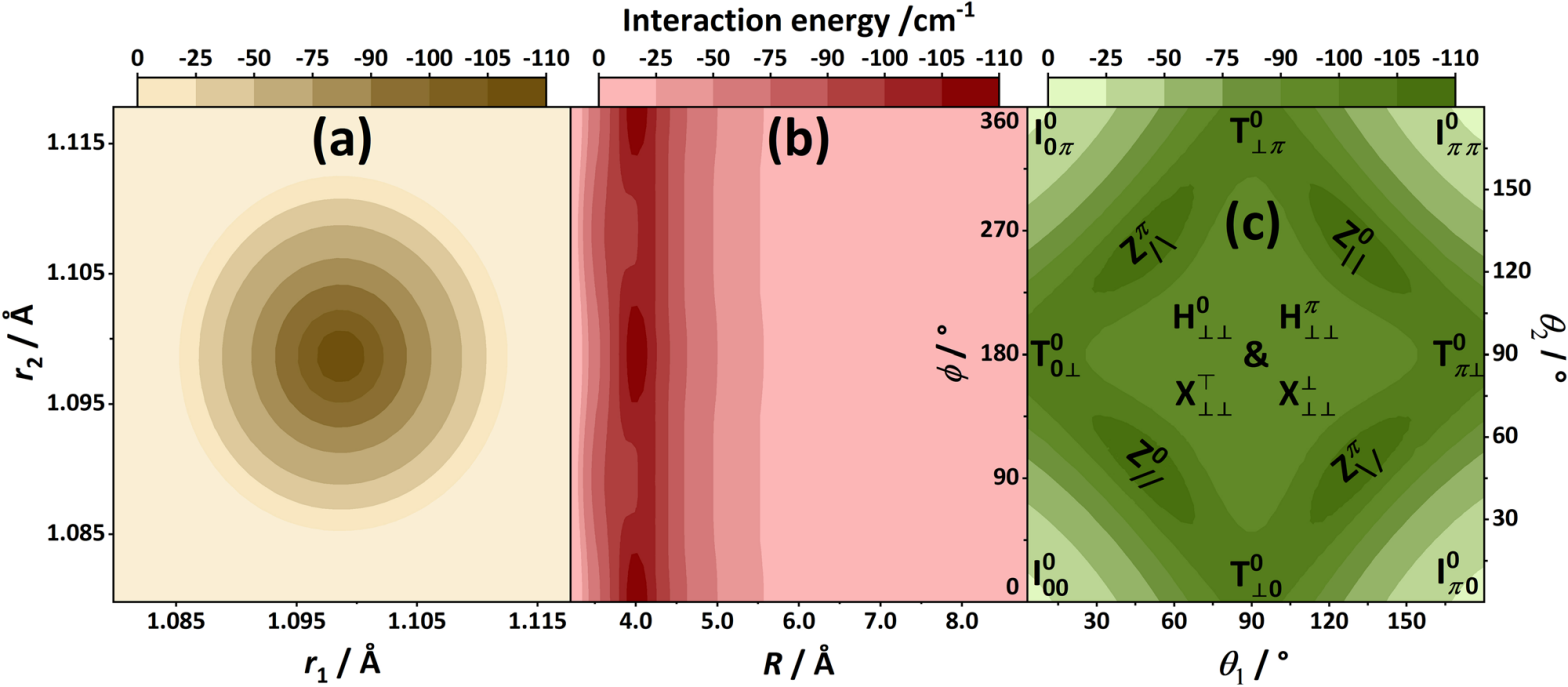
Contour plots determined from two-dimensional (2D) scans of the N2d-CC potential energy surface (PES). For each 2D point, the remaining four variables were fully relaxed. The contour levels represent interaction energies, with scales given separately on the top of each panel. The energetically equivalent Z-, T-, H-, X-, and I-shaped forms are shown explicitly in panel (c), following the ${S}_{{\theta }_{1}{\theta }_{2}}^{\phi }$ notation for a shape *S*, where the specific angle values are / ≈ 49. 5°, ⊥ ≡ 90°, \ ≈ 130. 5°, *π* ≡ 180°, and ⊤ ≡ 270°.

According to the interaction energies given in Fig. 2, the Z-shaped global minimum of the N_2_ dimer lies below the T-shaped transition state by only roughly 6 cm^−1^. Moreover, two other salient, higher-order saddle points, X and H, are also energetically similar to the Z-shaped minimum. This suggests that the PES is shallow over a large part of the interacting regime. To gain deeper insight into the overall topology of the PES in the most important regions, contour plots have been constructed for the (*r*_1_, *r*_2_), (*R*, *ϕ*), and (*θ*_1_, *θ*_2_) coordinate pairs (see the three panels of Fig. 4).

As expected and apparent from Fig. 4(a), the energy increases sharply when the *r*_1_ and *r*_2_ coordinates describing the two monomer stretches are distorted. To help interpret Fig. 4(a), recall the minuscule Δ*r*_1_ and Δ*r*_2_ values of Fig. 2 and the small shifts and splittings displayed in Table 2. As seen in Fig. 4(b), the *R* variable, that is the dimer’s vdW dissociation coordinate, is relatively tightly bound at its equilibrium value, ~4.0 Å. In clear contrast, motion along the torsional coordinate *ϕ* is extremely soft, without considerable barriers over the complete [0, 2*π*) angular range, as shown in Fig. 4(b). As obvious from Fig. 4(c), the Z-shaped form can also distort quite easily along the two bending coordinates, there is no pronounced angular preference in the broad, dark-green domain. These observations leave no doubt that the N_2_ dimer complies with criterion (i) of quasistructurality.

#### Heavily mixed vibrational states

**Fig. 5:**
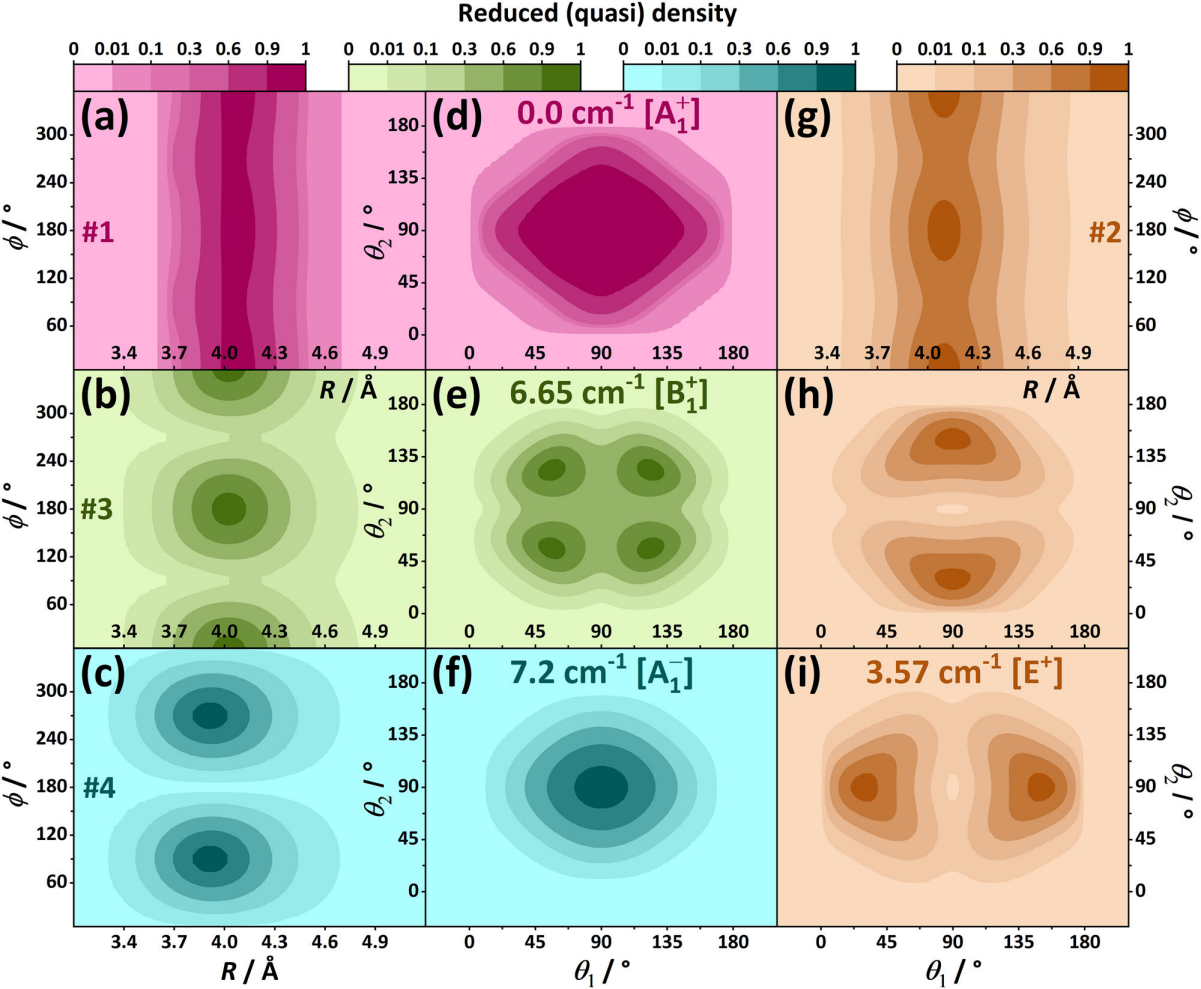
Probability-density distributions for the first four vibrational states of the ^14^N_2_⋅^14^N_2_ complex. Panels with identical background color refer to the same state. The #*n* symbol is the unique serial number of a vibrational state (in an increasing energy order, counting the *E*^+^ state once). The symmetry labels, as well as the quantum-state energies are indicated once for each state. The (*R*, *ϕ*) distribution is identical for the two components of the *E*^+^ state. The reduced (quasi) density is calculated as the sum of the squared eigenvector entries (at high grid resolution) for a specific 2D point, without reliance on the quadrature weights.

To gain insight into the vibrational characteristics of N_2_⋅N_2_, probability densities have been generated for all the bound *J* = 0 states of ^14^N_2_⋅^14^N_2_, as well as for its two intramonomer stretch fundamentals. Density distributions of the first four vibrational states, of ${A}_{1}^{+}$, *E*^+^, ${B}_{1}^{+}$, and ${A}_{1}^{-}$ symmetry, in order, are shown in Fig. 5 (plots of the other *J* = 0 states are available in an external repository^86^). Since the (*r*_1_, *r*_2_) graphs exhibit a simple and regular ground-state density profile for the bound states, without noticeable variations, in Fig. 5 density distributions are given solely for the (*R*, *ϕ*) and (*θ*_1_, *θ*_2_) subspaces. Supplementary Fig. 1 provides a density-based comparison between the ground state and the two intramonomer fundamentals, where the (*r*_1_, *r*_2_) plots are also included.

In accordance with the flatness of the interaction PES along the three angular coordinates (see Section “Extremely flat interaction PES”), the density distributions of Fig. 5 show that the nuclei of N_2_⋅N_2_ are prone to delocalization along all three of them. This is true even for the ground vibrational state, where (a) along the torsional coordinate *ϕ*, at fixed *R* values the density does not vary noticeably [see Fig. 5(a)], and (b) the points with high densities are accumulated in the broad and square-shaped central part of Fig. 5(d). These quasi-isotropic density motifs suggest that the effective ground-state structure of N_2_⋅N_2_ is qualitatively dissimilar to the planar, Z-shaped global minimum. These non-trivial features observed imply in themselves that the N_2_⋅N_2_ dimer fulfills quasistructurality criteria (ii) and (iii).

In panel Fig. 4(c), the four minimum-like features correspond to the four versions^79^ of the planar Z-shaped form of the ^14^N_2_⋅^14^N_2_ dimer. This suggests that our understanding of the complicated energy and density patterns can be enhanced by a model calculation which artificially localizes the vibrational eigenfunctions around one of the four equivalent Z-shaped versions. In this ‘artificial localization model’, whose precise definition is provided in Section “Methods”, each vibrational state becomes four-fold degenerate.

**Fig. 6:**
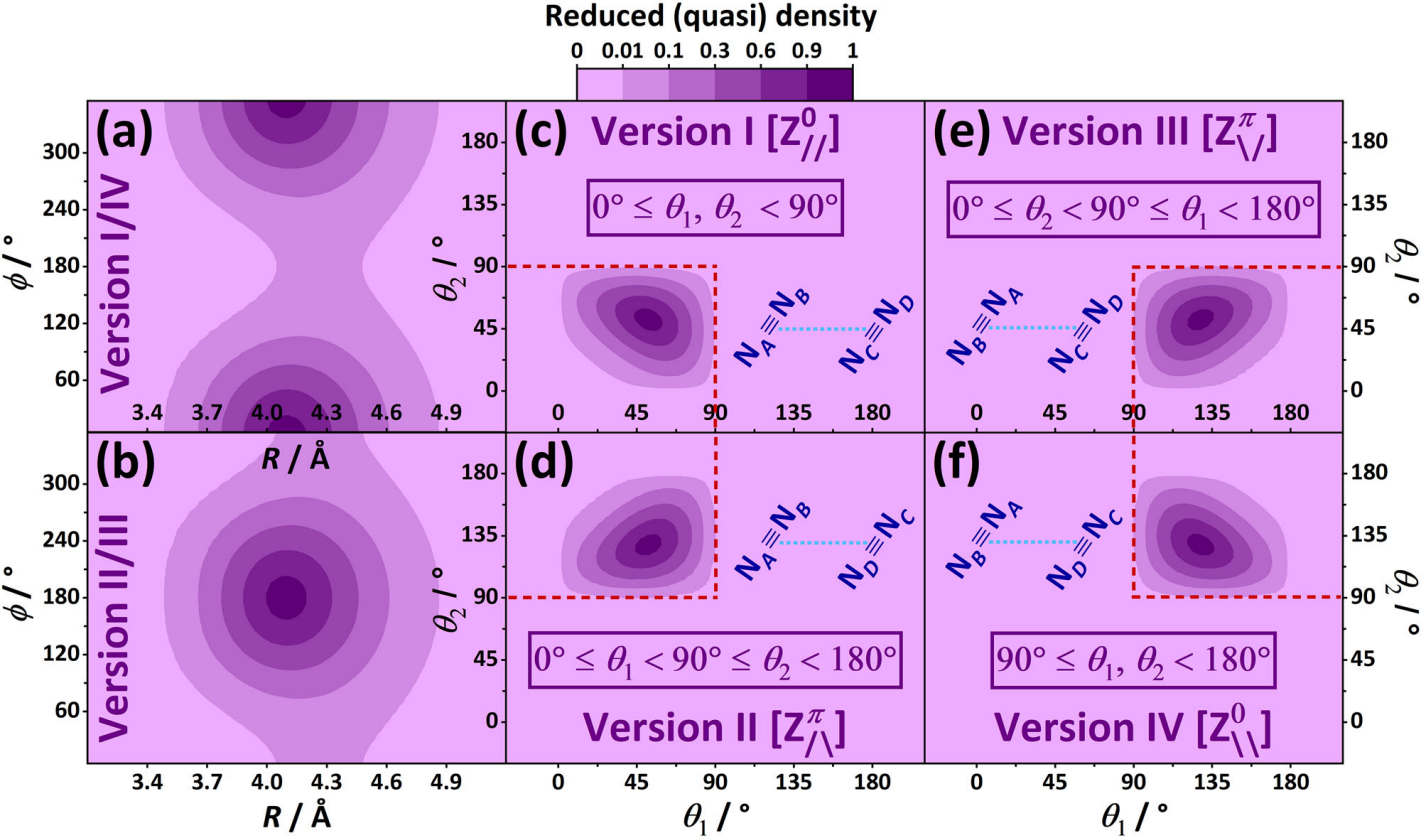
Ground-state density distributions derived for the artificial localization model. **a**, **b** and **c**–**f** show the (*R*, *ϕ*) and (*θ*_1_, *θ*_2_) localized density plots of the four Z-shaped versions, respectively. The naming convention applied for these versions are the same as in Fig. 4. For specific details about these plots, consult the caption to Fig. 5. The densities of these four versions are localized in coordinate regions specified in the violet boxes. Note that these versions with the same torsion angle, namely ${{{\rm{Z}}}}_{//}^{0}$ & ${{{\rm{Z}}}}_{\backslash\backslash}^{0}$ and ${{{\rm{Z}}}}_{\backslash/}^{\pi}$ & ${{{\rm{Z}}}}_{/\backslash}^{\pi}$, are characterized by identical (*R*, *ϕ*) density plots, as seen in (**a**, **b**), respectively. The schematic representation of the four Z-shaped versions is given in dark blue on (**c**–**f**), where N_*A*_ ≡ N_*B*_ and N_*C*_ ≡ N_*D*_ denote the monomers.

The ground-state density distributions of the localized states of the four Z-shaped versions are shown in Fig. 6. In accordance with the torsion angles, the (*R*,  *ϕ*) densities are concentrated around *ϕ* = 0° [Fig. 6(a)] and *ϕ* = 180° [Fig. 6(b)] for versions I/IV and II/III, respectively. As expected, the artificial localization model gives much simpler density patterns than those seen in Fig. 5. This holds for the excited vibrational states, as well, whose density distributions are not given here.

**Fig. 7:**
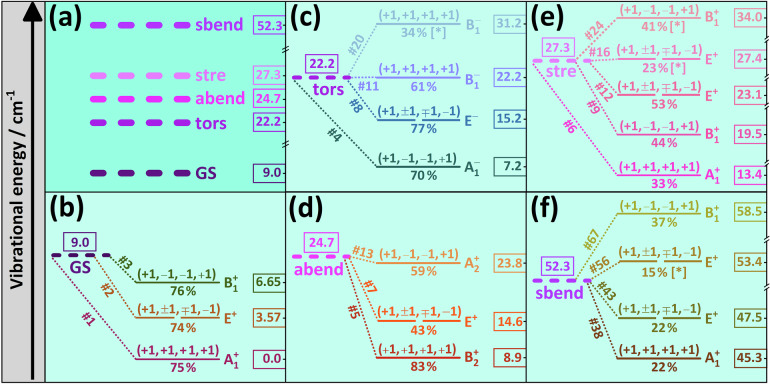
Correspondence of the vibrational states of ^14^N_2_⋅^14^N_2_ with those of the artificial localization model. **a** shows the energies of five vibrational states of the artificial localization model of Section “Models”. These artificially localized states correspond to the ground vibrational state (GS), the torsional (tors), the antisymmetric bend (abend), the intermonomer stretch (stre), and the symmetric bend (sbend) fundamentals. **b**–**f** display each vibrational state of ^14^N_2_⋅^14^N_2_ whose wave function expansion, specified in Eq. (7), contains the largest contribution from one of the states of (**a**). Symbol #*n* has the same meaning as in Fig. 5. In each panel, the energy values are placed into colored boxes. These vibrational energies, given in cm^−1^, are relative to that of state #1 in (**b**). The individual states are illustrated with horizontal lines, where the number of dashes corresponds to their degeneracy factor. The states are distributed according to the right-hand-side vertical energy axes (note the several axis breaks). A sign quadruplet, *e.g*., (+1, +1, +1, +1) for the GS, serves as an assignment of a vibrational state, and contains the signs attached to the most dominant contribution according to Eq. (7). The squared coefficients of these dominant terms are given as percentages. The asterisks in brackets highlight the “extra” states which break the four-fold overall degeneracy of the artificial localization model.

Figure 7 (a) shows the energy-level structure of the artificial localization model, where the energies are relative to the true vibrational ground state of ^14^N_2_⋅^14^N_2_. As apparent from Fig. 7(b), the first three vibrational states of ^14^N_2_⋅^14^N_2_, of ${A}_{1}^{+}$, *E*^+^, and ${B}_{1}^{+}$ symmetry, are reproduced, with a squared overlap of  ≈ 75 %, by the signed sums of the four artificially localized ground-state eigenfunctions (the remaining 25 % comes from several excited vibrational states). Basically the same holds for the antisymmetric bend shown in Fig. 7(d), with symmetry species ${B}_{2}^{+},{E}^{+}$, and ${A}_{2}^{+}$. This simple model is unable to explain Fig. 7(c), (e), and (f), whereby further states, indicated with bracketed asterisks, mix in. Excluding these extra states from consideration, the retained state triplets fully reproduce the degeneracy factor of the states illustrated in Fig. 7(c), (e), and (f). Overall, these results yield clear evidence that quasistructurality criteria (iii) and (iv) are satisfied for N_2_⋅N_2_.

#### Strong rovibrational couplings

**Fig. 8:**
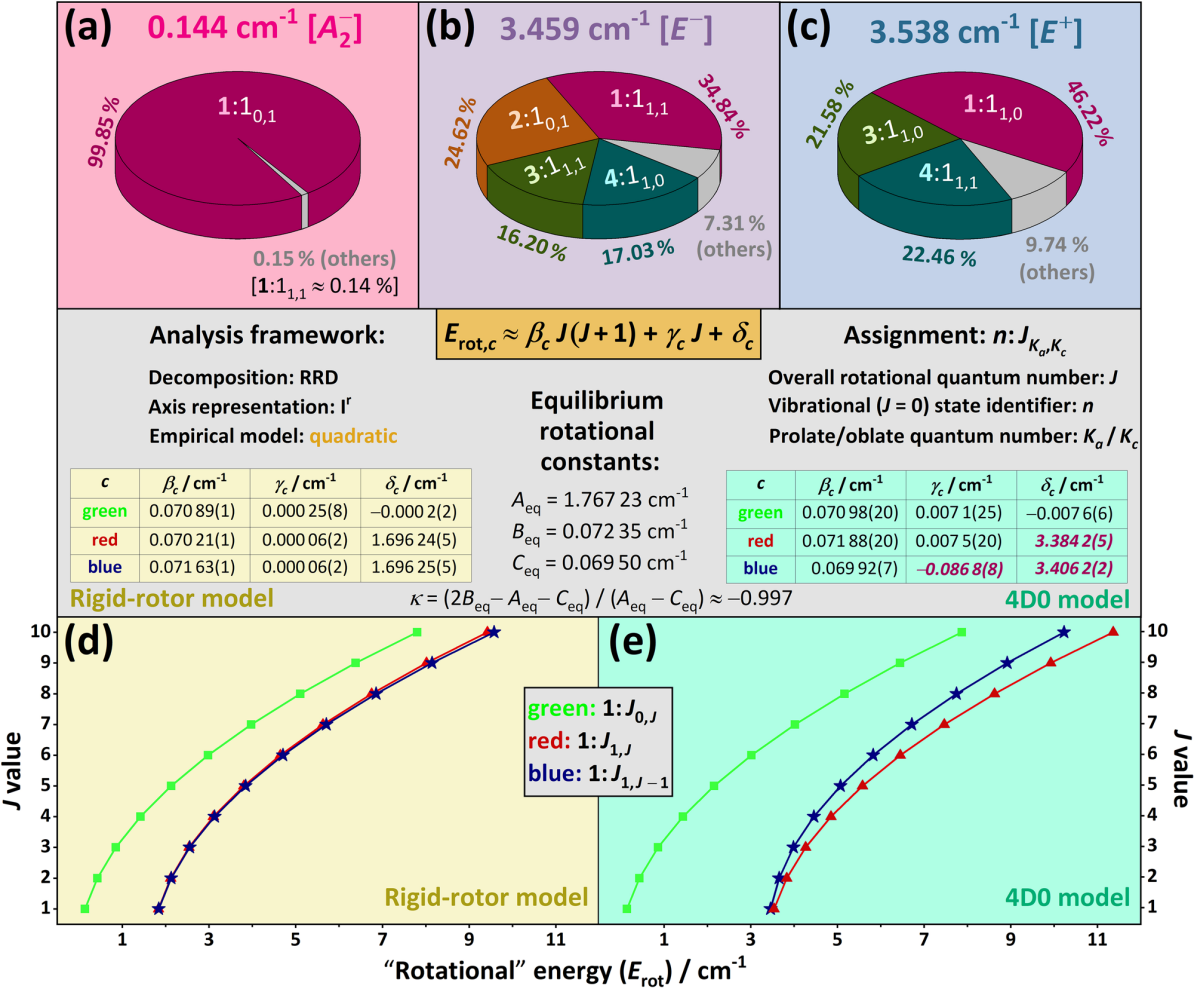
Illustration of the strong rovibrational couplings in selected rovibrational states of the ^14^N_2_ ⋅ ^14^N_2_ complex. The pie charts of (**a**–**c**) demonstrate the results obtained for the lowest three *J* = 1 states, using the rigid-rotor decomposition (RRD) method^95^ and the I^r^ axis representation^120^. The percentages on (**a**–**c**) are squared overlaps among the true eigenstates and the products of the pure vibrational (*J* = 0) and the rigid-rotor (RR) eigenstates [the assignments of these products are defined in the central panel]. The RRD contributions coming from the two eigenvectors of the degenerate eigensubspaces are summed up in the percentages. Notice that the same colors are used here and in Fig. 5 to denote the vibrational states. For all the bound rovibrational states computed during this study, the mixing coefficients form part of an external repository^86^. **d**, **e** describe the *J* dependence of the lowest three rovibrational energies in the RR and the variational (4D0) models, respectively (for simplicity, the horizontal and vertical axes are swapped). For these two datasets, a quadratic model was fitted, whose parameters, along with their last-few-digit (two-sigma) uncertainties in parentheses are given in the central panel. The rotational constants of the Z-shaped global minimum, derived from the N2d-CC PES, as well as its Ray asymmetry constant *κ*^79^, are also reported in the central panel. Those fitted parameters of the 4D0 dataset which differ considerably from their RR counterparts are typeset in purple bold italics.

For semirigid molecules, separation of the vibrational and rotational degrees of freedom works extremely well for at least the ground vibrational state and the fundamentals, as the vibrational and rotational excitation energies are drastically different, often by more than an order of magnitude. This is not true for the N_2_ dimer. The lowest-lying bound vibrational (*J* = 0) states of ^14^N_2_⋅^14^N_2_ have energies of only a few cm^−1^, and there are four excited vibrational states with energies less than 10 cm^−1^ [see Fig. 7(b)–(d)]. Therefore, these vibrational states can couple effectively with the rotational states, as *A*_eq_ is close to 2 cm^−1^ for ^14^N_2_⋅^14^N_2_ (for the three rotational constants of the Z-shaped global minimum, as well as the other symbols used in this subsection, see the central panel of Fig. 8). Extremely strong coupling between vibrations and rotations, starting at the lowest rotational excitation, has been identified in several neutral and charged molecular systems^80,88–94^. As shown next, similar interactions, which are signs of quasistructural behavior, are present in ^14^N_2_⋅^14^N_2_, as well.

Figure 8 shows a collection of rovibrational states with low *J* and *K*_*a*_ values, which were subjected to a rigid-rotor decomposition (RRD) analysis^95^, where *K*_*a*_ designates the approximate prolate-top rotational quantum number of the asymmetric-top ^14^N_2_⋅^14^N_2_ dimer. As clear from Fig. 8(a), the rigid-rotor model holds very well for the lowest *J* = 1 state of ${A}_{2}^{-}$ symmetry, as *B*_eq_ + *C*_eq_ = 0.142 ≈ 0.144 cm^−1^, the energy of this state. Thus, the parent vibrational state of this *J* = 1 rovibrational state can be safely given as the ground vibrational state. On the other hand, Fig. 8(b) and (c) show the breakdown of the rigid-rotor model, as more than half of the vibrational contributions come from excited vibrational states for both the second and third *J* = 1 rovibrational states at 3.459 and 3.538 cm^−1^, respectively. For the rotational energies of these formally “1_1,1_” [*E*^−^] and “1_1,0_” [*E*^+^] states, the rigid-rotor model provides significantly different values, with *A*_eq_ + *C*_eq_ = 1.837 cm^−1^ and *A*_eq_ + *B*_eq_ = 1.839 cm^−1^, respectively. Thus, in the case of ^14^N_2_⋅^14^N_2_, the formal separation of the vibrational and rotational degrees of freedom leads to qualitatively incorrect energies and unacceptable assignment attempts even in the case of the lowest rovibrational states. These are clear signs of quasistructural behavior.

The bottom two panels of Fig. 8 demonstrate how the rotational energies vary, as a function of the *J* values, in the rigid-rotor and the variational 4D0 models (note the inverted vertical and horizontal axes). As to the *J*_0,*J*_ states [green curves in Fig. 8(d) and (e)], the results of the two models follow the same quadratic polynomial trend (see the central panel of Fig. 8 for the parameters of the fitted polynomial). As to the *J*_1,*J*−1_ and *J*_1,*J*_ states, the trends are still quadratic, but the linear and constant terms are considerably different for the two datasets.

With a Ray asymmetry parameter of *κ* ≈ − 0.997 (see the central panel of Fig. 8), the equilibrium structure of ^14^N_2_⋅^14^N_2_ corresponds to a nearly prolate symmetric top; thus, the rigid-rotor model predicts fairly small splittings for the *J*_1,*J*−1_ and *J*_1,*J*_ states. In contrast, the variationally computed splittings significantly grow towards larger *J* values, indicating increased rovibrational coupling as *J* increases. While the symmetric-top quantum number *K*, which corresponds to *K*_*a*_ for prolate symmetric tops, may behave as a nearly good quantum number even for vdW dimers^96,97^, this is seemingly not the case for (N_2_)_2_.

As shown in Fig. 8(b), there is considerable coupling between the *K*_*a*_ = 0 and *K*_*a*_ = 1 rigid-rotor eigenfunctions even for *J* = 1, a feature documented neither in ref. ^66^ nor in refs. ^77^ and ^78^. For further insight into the rovibrational mixings present in ^14^N_2_⋅^14^N_2_, see the RRD files given in an external repository^86^. In the *J* = 3 case, where most of the states exhibit pronounced *K*_*a*_ mixings, the RRD coefficients have been determined using both the N2d-CC and the N2d-H0B PESs, revealing typical deviations on the order of only 1–2 %. This means that the mixing appears to be a genuine physical effect rather than an artifact due to the analytical PESs employed.

Performing a global fit to all of the *J *≤ 10 bound rovibrational states where the ground vibrational state gives the dominant RRD contribution, the following empirical model, not shown in Fig. 8, was obtained (for improved transparency, the  ± notation is used here to display the two-sigma uncertainties of the parameters):

2 $${E}_{{{\rm{rot}}}}(J,{K}_{a})\,/\,{{{\rm{cm}}}}^{-1}= 	 \, (0.071\pm 0.002)\,J(J+1) +(1.95\pm 0.02)\,{K}_{a}^{2} \\ 	 +(1.5\pm 0.1)\,{K}_{a}.$$

Note the importance of the inclusion of the term linear in *K*_*a*_ in the energy expression. In our previous studies on CH_4_ ⋅ F^-^^88^ and ${{{\rm{H}}}}_{5}^{+}$^89^, due to the strong coupling between the vibrational and rotational degrees of freedom, a term linear in *K* was also shown to have a remarkable impact on the rotational-vibrational energy level pattern. Clearly, the discussion presented above about the various aspects of the coupling of rotations and vibrations provides further evidence that N_2_⋅N_2_ satisfies quasistructurality criteria (iii) and (v).

## Concluding remarks

Non-covalent interactions govern the dynamical behavior and properties of numerous molecular systems; thus, their detailed understanding is of extreme importance when studying weakly-bound molecular systems, however small or large these interactions are. In this work, the structure, nuclear dynamics, and rovibrational states of the prototypical van der Waals (vdW) dimer, N_2_⋅N_2_, have been investigated. Our computational study relied on two newly created full-dimensional potential energy surfaces, designed with spectroscopic (≈ 1 cm^−1^) accuracy in mind.

Symmetry-adapted variational solution of the nuclear Schrödinger equation resulted in some 6000 bound rovibrational states for the parent ^14^N_2_⋅^14^N_2_ isotopologue up to *J* = 10, where *J* is the rotational quantum number. Following the recommendations of ref. ^52^ and a recent review on reporting spectroscopic data^98^, individual uncertainty estimates, with a median value of 0.8 cm^−1^, are also reported for each state, along with symmetry labels corresponding to the irreducible representations of the *G*_16_ molecular symmetry group. Accurate shifts and splittings have been determined for the quasi-bound intramonomer stretch fundamentals, as it is expected that the yet missing experimental results can be most easily generated in this spectral region. Isotope effects have also been considered for the lowest excited vibrational states of N_2_⋅N_2_, where the shifts and splittings computed were perfectly explained by a simple bilinear model.

Based on the extensive computational results of this study and the thorough analysis of the intriguing structural/dynamical features observed, it has been firmly established that N_2_⋅N_2_ is a quasistructural species, whereby, for example, considerable mixing can be observed in the lowest-energy rovibrational quantum states among the four versions of its Z-shaped global minimum. Similar vdW dimers are expected to fall into the class of quasistructural systems, meaning that the interpretation of their observed high-resolution spectra would require a special view of their nuclear motion and molecular structure.

## Methods

### Internal coordinates and nuclear-motion models

During the present study, the nuclear motions of the nitrogen dimer, N_2_⋅N_2_, have been represented with the six curvilinear internal coordinates given in the first two columns of Table 4, whereby the functions stre( ⋅ ,  ⋅ ), bend( ⋅ ,  ⋅ ,  ⋅ ), and tors( ⋅ ,  ⋅ ,  ⋅ ,  ⋅ ) follow the conventional definitions of bond lengths, bond angles, and torsion angles, respectively^99,100^. This internal-coordinate system is ill-defined for *θ*_2_ ∈ {0, *π*}: in that case, *ϕ* is arbitrary and thus one can set *ϕ* = 0. The last four columns of Table 4, under ‘Model’, contain information about the utilization of the internal coordinates during the solution of the time-independent nuclear-motion Schrödinger equation (see Section “Symmetry-adapted variational nuclear-motion computations”). The 6D, 4D, 4D0, and 2D models are defined by a collection of active/frozen (A/F) coordinates, as well as by the values where the frozen coordinates are fixed.

**Table 4:** The internal coordinates adopted in this study to describe the molecular structure of the N_2_ dimer^〈*a*〉^

Internal coordinate			DVR		Model^〈*g*〉^			
Label	Definition^〈*b*〉^	Range^〈*c*〉^	Type^〈*e*〉^	Size^〈*f*〉^	6D	4D	4D0	2D
*r*_1_	stre$({{{\rm{N}}}}_{1}^{{\prime} },{{{\rm{N}}}}_{1}^{{\prime\prime} })$	[0.9 Å, 1.3 Å]	PO-Laguerre^121,122^	6/6	A	F{Z}	F{Z0}	A
*r*_2_	stre$({{{\rm{N}}}}_{2}^{{\prime} },{{{\rm{N}}}}_{2}^{{\prime\prime} })$	[0.9 Å, 1.3 Å]	PO-Laguerre^121,122^	6/6	A	F{Z}	F{Z0}	A
*R*	stre(COM_1_, COM_2_)	[3.3 Å, 8.4 Å]^〈*d*〉^	Hermite^122^	45/6	A	A	A	F{Z}
*θ*_1_	bend$({{{\rm{N}}}}_{1}^{{\prime\prime} },{{{\rm{COM}}}}_{1},{{{\rm{COM}}}}_{2})$	(0, *π*)	cotangent^117^	14/12	A	A	A	F{Z}
*θ*_2_	bend$({{{\rm{N}}}}_{2}^{{\prime} },{{{\rm{COM}}}}_{2},{{{\rm{COM}}}}_{1})$	(0, *π*)	cotangent^117^	14/12	A	A	A	F{Z}
*ϕ*	tors$({{{\rm{N}}}}_{1}^{{\prime} },{{{\rm{COM}}}}_{1},{{{\rm{COM}}}}_{2},{{{\rm{N}}}}_{2}^{{\prime} })$	[0, 2*π*)	exponential^123–125^	12/8	A	A	A	F{Z}

^〈*a*〉^The coordinates are defined for a distinguished version^79^ of the N_2_ dimer, with its two monomers ${{{\rm{N}}}}_{1}^{{\prime} }\equiv {{{\rm{N}}}}_{1}^{{\prime\prime} }$ and ${{{\rm{N}}}}_{2}^{{\prime} }\equiv {{{\rm{N}}}}_{2}^{{\prime\prime} }$, whose centers of mass are COM_1_ and COM_2_, respectively. These sites specify the right-handed embedding chosen in this study, where (i) $\overleftarrow{{{{\rm{COM}}}}_{2}\,{{{\rm{COM}}}}_{1}}$ is the direction vector of the *z* axis, (ii) ${{{\rm{N}}}}_{1}^{{\prime} }$ lies on the positive *x*-side of the *xz* plane, and (iii) the origin is shifted to the dimer’s COM.

^〈*b*〉^The definition of the internal coordinates uses the standard abbreviations “stre”, “bend”, and “tors”, corresponding to stretching, bending, and torsional coordinates, respectively^100^, which depend on the positions of the sites listed in parentheses.

^〈*c*〉^Coordinate ranges applied for the coordinates, optimized for the variational nuclear-motion computations of this study.

^〈*d*〉^For the two intramonomer stretch fundamentals, the *R* range could be reduced to [3.6, 4.7] Å.

^〈*e*〉^Discrete variable representation (DVR) basis types applied during our first-principles nuclear-motion computations. The prefix “PO-” means that potential optimization^121^ was employed for the underlying grid points with the help of a 1D model.

^〈*f*〉^Optimal DVR sizes for the bound/resonance states, separated by slashes.

^〈*g*〉^Dynamical models with active/frozen coordinates designated as A/F. Coordinates with “F{Z}” are frozen at their values within the Z-shaped global minimum. In the 4D0 model, “F{Z0}” means that a vibrationally averaged N  ≡ N ("*r*_0_”) bond length is used for the fixed coordinates rather than the equilibrium bond lengths of the Z-shaped form. To obtain *r*_0_ values for the two new PESs, a vibrational correction, estimated as *δr*_0_ = 0.0037 Å^72^, was added to the equilibrium bond lengths.

### Electronic-structure computations

The electronic-structure computations of this study have been performed at the Hartree–Fock, MP2, SAPT, and coupled-cluster levels (up to perturbative pentuple excitations in the latter case), utilizing Dunning’s aug-cc-pV*X*Z basis sets^101^ up to *X* = 6. These computational results form the basis of an FPA analysis^49,51^, and they helped to construct two full-dimensional PESs for the N_2_ dimer at the “CC” and “SAPT” levels (see Table 5). The “SAPT” protocol corresponds to a density-fitted SAPT(DFT) scheme^102–104^, where the PBE0^105^ functional is adopted, alongside the aug-cc-pVQZ basis set^101^, to describe the electronic structure of the isolated N_2_ monomer. The “CC” approach represents counterpoise-corrected, frozen-core CCSD(T), where a CBS extrapolation is carried out using the aug-cc-pVTZ and aug-cc-pVQZ basis sets^101^ plus midbond functions^106^. Within the “CC” scheme, the Hartree–Fock and the correlation terms were extrapolated *via* formulas reported in refs. ^94^ and ^107^, respectively.

**Table 5:** The main characteristics of the two full-dimensional potential energy surfaces developed during this study^〈*a*〉^

Indicator	N2d-SAPT	N2d-CC
${({z}_{1}^{{{\rm{OA}}}},{z}_{2}^{{{\rm{OA}}}},{z}_{3}^{{{\rm{OA}}}},{z}_{4}^{{{\rm{OA}}}},{z}_{5}^{{{\rm{OA}}}})}^{\langle b\rangle }$	(–0.436, –0.219, 0, 0.219, 0.436)	(–0.676, –0.272, 0, 0.272, 0.676)
range(*r*_1/2_)^〈*c*〉^	[–0.08, 0.10] + 1.088 83^〈*d*〉^	[–0.09, 0.09] + 1.098 66^〈*d*〉^
range(*R*)^〈*c*〉^	[2.5, 8.4]	[2.7, 8.4]
range(Δ*E*_int_)^〈*e*〉^	[–104.8, 6499]	[–107.4, 9181]
range(Δ*E*_inter_)^〈*e*〉^	[–106.2, 3683]	[–107.4, 5127]
range(Δ*E*_intra_)^〈*e*〉^	[0, 6200]	[0, 9182]
${N}_{{{\rm{par}}}}^{\langle f\rangle }$	113	143
${N}_{{{\rm{geo}}}}^{\langle g,i\rangle }$	171 / 2263 / 2437	172 / 1699 / 1847
RMSD^〈*h*, *i*〉^	0.48 / 0.64 / 0.62	0.18 / 0.23 / 0.97

^〈*a*〉^The parameters listed here pertain to the final version of the two (N2d-SAPT and N2d-CC) potentials, relying on a new sampling scheme described in Supplementary Note 2.

^〈*b*〉^Positions of the five off-atomic (OA) sites, specified in Å, alongside the *z* axis; that is, the symmetry axis of the isolated N_2_ monomer, whose center of mass (COM) corresponds to (*x*, *y*, *z*) = (0, 0, 0).

^〈*c*〉^Ranges of the radial (*r*_1_ and *r*_2_, and *R*) coordinates within the fitting dataset, given in Å.

^〈*d*〉^Equilibrium N ≡ N bond lengths of the isolated N_2_ molecule derived from the two PESs.

^〈*e*〉^Ranges of the interaction energies and their (intra/inter)monomer contributions, in cm^−1^, within the fitting dataset.

^〈*f*〉^Number of parameters used in the intermonomer fit. Beyond them, 13 parameters were also employed in the intramonomer fit to reproduce the Rydberg–Klein–Rees potential of the isolated N_2_ monomer.

^〈*g*〉^Number of dimer geometries generated by the sampling process for the intermonomer fit.

^〈*h*〉^Root-mean-square deviations (RMSD), in cm^−1^, characterizing the intermonomer fit.

^〈*i*〉^These rows comprise data triplets separated by slashes, representing two distinguished subsets of grid points, subsets I and II, and the whole grid set. Subset I contains geometries with negative interaction energies, while subset II includes those configurations whose probability-density-based weights are not smaller than 0.1 [for this weighting scheme, see Supplementary Note 2].

The comparisons given in Fig. 1 are based on Hellmann’s direct electronic-structure computations^72^. Hellmann published reference (intermonomer) interaction energies for 408 dimer configurations, keeping the two intramonomer distances fixed at the vibrationally averaged (“*r*_0_”) bond length of the isolated N_2_ molecule. Among the different levels considered by Hellmann, the highest one is what is abbreviated here as H0B ( ≡ Hellmann’s *r*_0_-based benchmark scheme), which includes perturbative quadruples [(Q)], counterpoise, core-core plus core-valence, and relativistic corrections near the complete basis set (CBS) and full configuration interaction (FCI) limits^33,53^. The (Q) correction is indeed the highest-order coupled-cluster term which can be afforded for hundreds of dimer arrangements using a reasonable basis set. In the H0B energy values, Hellmann applied a scaling factor of 0.5 for the (Q) corrections to increase the accuracy of virial coefficients yielded by his quasiclassical Monte-Carlo simulations. It turns out, however, from our examination, that the post-CCSD(T) effects do not play a significant role in achieving spectroscopic (≈1 cm^−1^) accuracy for the rovibrational states of N_2_⋅N_2_: this justifies the neglect of the expensive iterative triples and (Q) terms during the determination of the full-dimensional (6D) PESs.

### PES construction

To determine 6D PESs for the N_2_ dimer, the autoPES program suite^35,39^, interfaced to SAPT^58^, MOLPRO^108^, and ORCA^109^, was employed, ensuring a highly automatic treatment. To help evaluate the uncertainty of our rovibrational results, two PES versions, named N2d-SAPT and N2d-CC, were created with autoPES, corresponding to the “SAPT” and “CC” levels, respectively (for the specifications of these two computational protocols, see Section “Potential energy surfaces and stationary points”). Concerning the final versions of these two PESs, a few important characteristics, those that guide our description of their generation, are listed in Table 5.

To enhance the flexibility of our fitted 6D PESs, five off-atomic (OA) sites were introduced for both monomers: one at its center of mass (COM) and two others on the two sides of the COM along the principal symmetry axis. The OA positions were optimized with the PES parameters, forcing them to preserve the monomer’s *D*_*∞h*_ point-group symmetry. Permutation invariance of the PESs was maintained by introducing a common set of parameters for the four nitrogen atoms of the N_2_ monomers. Under these constraints, the interaction energies were treated as the sums of the intramonomer (deformation) and intermonomer energies:

3 $$\Delta {E}_{{{\rm{int}}}}({r}_{1},{r}_{2},{{\boldsymbol{\rho }}})=\Delta {E}_{{{\rm{intra}}}}({r}_{1},{r}_{2})+\Delta {E}_{{{\rm{inter}}}}({r}_{1},{r}_{2},{{\boldsymbol{\rho }}}),$$

where ${{\boldsymbol{\rho }}}={(R,{\theta }_{1},{\theta }_{2},\phi )}^{{{\rm{T}}}}$ designates the intermonomer ("relative”) coordinates of the N_2_ dimer, with the restriction that Δ*E*_intra_(*r*_e_, *r*_e_) = 0 and ${\lim }_{R\to \infty }\Delta {E}_{{{\rm{inter}}}}({r}_{1},{r}_{2},{{\boldsymbol{\rho }}})=0$ (*r*_e_ symbolizes the equilibrium bond length of the isolated N_2_ molecule at the “CC”/"SAPT” level). The term Δ*E*_inter_(*r*_1_, *r*_2_, ***ρ***) is based on damped interaction (exponential, Coulombic, induction + dispersion, and polarization) models^35,39^, while the Δ*E*_intra_(*r*_1_, *r*_2_) contribution is fitted as a sum of two-body polynomials^39^ in a separate phase, trained on “experimental” (Rydberg–Klein–Rees) deformation-energy values^1,110^ (see also Supplementary Note 2).

The intermonomer fitting process was divided into two parts, based on separate short- (*R* ≤ 7 Å) and long-range (*R* > 7 Å) configuration spaces. In the short-range regime, the intermonomer energies of the grid points were determined at the “SAPT” and “CC” levels. In the long-range domain, where the accuracy requirement is less stringent, the intermonomer energies were computed for 9000 grid points with a multipole expansion along the *R* coordinate (see refs. ^35^ and ^111^). To produce preliminary versions for the N2d-SAPT/CC PESs, the standard built-in algorithms of the autoPES code were employed during the grid-generation and the PES-parametrization process (see Secs. II–IV of ref. ^39^). This procedure was performed according to an iterative (grid generation – fitting – identification of minima – hole fixing) scheme, until a reliable intermonomer fit was obtained.

After computing the two intramonomer stretch fundamentals for N_2_⋅N_2_, it has become apparent that the accuracy/stability of their splitting tends to be limited across the intermediate versions of the N2d-CC/SAPT PESs. This may be due to the fact that autoPES focuses mostly on the lower-energy (Δ*E*_int_ < 0) region of the “CC”/"SAPT” PES while sampling the grid points and weighting the fitted data. Thus, a new grid-sampling and weighting scheme has been implemented in autoPES, using direct-product-based wave-function coefficients to define point-by-point weights (this method is akin to density-guided PES sampling advocated in ref. ^112^). A couple of important details about this new protocol, called amplitude-driven sampling (ADS), are provided in Supplementary Note 2. These modified weights, in combination with ADS, led to accurate N2d-CC/SAPT PESs, enabling us to compute (a) rovibrational energies for N_2_⋅N_2_ with an uncertainty of 0.5–1.5 cm^−1^, and (b) the shifts/splittings of the N ≡ N stretch fundamentals with an accuracy of  ±  0.1 cm^−1^ (for details, see Section “First-principles rovibrational results”).

### Symmetry-adapted variational nuclear-motion computations

When breaking the monomer bonds is not allowed, the quantum states of the N_2_⋅N_2_ isotopologues comprising four identical isotopes transform according to the irreducible representations (irreps) of the *G*_16_ molecular symmetry (MS) group^79,113^. The 16 distinct symmetry operations of the *G*_16_ group^79,113^ can be expressed as products of three elementary operations, ${{{\mathcal{E}}}}^{* }$, ${{{\mathcal{P}}}}_{1}$, and ${{{\mathcal{P}}}}_{12}$, where ${{{\mathcal{E}}}}^{* }$ is the space-inversion operation, ${{{\mathcal{P}}}}_{1}$ is a permutation within monomer 1, and ${{{\mathcal{P}}}}_{12}$ represents the permutation that interchanges the two monomers. The character table of the *G*_16_ group, with the ten irreps denoted as ${A}_{1/2}^{\pm }$, ${B}_{1/2}^{\pm }$, and *E*^±^, is given in Table A-25 of ref. ^79^ (note that our study uses the Merer–Watson convention^114^ for the irreps). For this group, the selection rules for the dipole-allowed transitions are ${A}_{1}^{\pm }\leftrightarrow {B}_{1}^{\mp }$, ${A}_{2}^{\pm }\leftrightarrow {B}_{2}^{\mp }$, and *E*^±^ ↔ *E*^∓^^66,78,113^. Note that the N_2_⋅N_2_ isotopologues containing non-identical nuclei belong to different subgroups of *G*_16_.

As nuclear spins are not considered explicitly during the solution of the time-independent nuclear Schrödinger equation, their effects must be taken into account *a posteriori*. Since ^14^N is a spin-1 nucleus, the monomers of the ^14^N_2_⋅^14^N_2_ isotopologue co-exist in separate *ortho* and *para* forms (with total nuclear spins 0/2 and 1, respectively). Thus, the rovibrational states of ^14^N_2_⋅^14^N_2_ can be divided into three sets: *ortho*–*ortho* (${A}_{1}^{+}$, ${A}_{2}^{-}$, ${B}_{1}^{-}$, and ${B}_{2}^{+}$), *para*–*para* (${A}_{1}^{-}$, ${A}_{2}^{+}$, ${B}_{1}^{+}$, and ${B}_{2}^{-}$), and *ortho*–*para* (*E*^+^ and *E*^−^)^66,78,113^. Since the nuclear-spin weights have nonzero values for all irreps of *G*_16_^113^, there are no missing symmetry blocks for ^14^N_2_⋅^14^N_2_ (in other words, all computed quantum states exist). The same holds for all the other isotopologues of the N_2_ dimer given in Table 3, with the exception of ^15^N_2_ ⋅ ^15^N_2_, where the ${A}_{2}^{-}$ and ${B}_{2}^{+}$ states do not exist.

Achieving converged rovibrational results required a large number of symmetry-adapted^92^ computations with the code GENIUSH^115,116^, used for the variational-like solution of the nuclear Schrödinger equation in both full and reduced dimensions. In these computations, the masses of the ^14^N and ^15^N nuclei were set to 14.003 074 and 15.000 109 u, respectively. The optimal discrete variable representation (DVR) basis sizes and radial-coordinate ranges, see Table 4, were selected so that all the vibrational energies reported are converged to within 0.02 cm^−1^ (for the two intramonomer stretch fundamentals, their shifts and splittings were also monitored, attaining a computational precision of 0.002 cm^−1^). Convergence of the two bending basis sets was significantly accelerated by using the cotangent DVR scheme^117^, which yields much smoother variations for the computed eigenvalues than the traditional Legendre-DVR basis^118,119^. Note that FBR (finite basis representation) basis sets exhibit better convergence for dimers of linear molecules^66,77,78^. In practice, though, FBR requires handling complicated, non-sparse potential-energy matrices, unlike DVR, where the potential matrix is diagonal. For further details, see Supplementary Note 3, as well as an external repository^86^, where the results of our convergence tests are placed in a folder called “conv_tests”.

Rovibrational energies have been computed with the aid of the following three PESs: N2d-H0B, N2d-SAPT, and N2d-CC. The computed energy values, *e*^[*J*]^, have an associated expanded (two-sigma) uncertainty, *U*^[*J*]^. The *e*^[*J*=0]^ energies are deduced from a 6D model, whose uncertainties are estimated as

4 $${U}^{[J = 0]}= 	 \, \left\vert {e}_{{{\rm{N}}}2{{\rm{d}}}\,{-}\,{{\rm{CC}}}}^{[J = 0]}(4{{\rm{D}}}0) - {e}_{{{\rm{N}}}2{{\rm{d}}}\,- \,{{\rm{H}}}0{{\rm{B}}}}^{[J = 0]}(4{{\rm{D}}}0)\right\vert \\ 	 +\left\vert \delta {e}_{{{\rm{N}}}2{{\rm{d}}}\,{\mbox{-}}\,{{\rm{CC}}}}^{[J = 0]}({{\rm{6D}}})-\delta {e}_{{{\rm{N}}}2{{\rm{d}}}\,{\mbox{-}}\,{{\rm{SAPT}}}}^{[J = 0]}({{\rm{6D}}})\right\vert ,$$

where ${e}_{p}^{[J]}(m)$ designates the rovibrational energy at a given *J* value, corresponding to PES version *p* and model dimensionality *m*, and $\delta {e}_{p}^{[J]}({{\rm{6D}}})={e}_{p}^{[J]}({{\rm{6D}}})-{e}_{p}^{[J]}(4{{\rm{D}}}0)$ is the 6D correction to the 4D0 rovibrational energy. For *J* > 0, only 4D0 computations have been made, leading to expanded uncertainties approximated as

5 $${U}^{[J\, > \, 0]}=\left\vert {e}_{{{\rm{N}}}2{{\rm{d}}}{\mbox{-}}{{\rm{CC}}}}^{[J\, > \, 0]}(4{{\rm{D}}}0)-{e}_{{{\rm{N}}}2{{\rm{d}}}{\mbox{-}}{{\rm{H}}}0{{\rm{B}}}}^{[J\, > \, 0]}(4{{\rm{D}}}0)\right\vert +{\alpha }_{\max }{e}_{{{\rm{N}}}2{{\rm{d}}}{\mbox{-}}{{\rm{CC}}}}^{[J\ > \ 0]}(4{{\rm{D}}}0),$$

where ${\alpha }_{\max }=0.64\, \%$ is the maximum value of an adjustment factor,

6 $$\alpha = 	 \left(\left\vert \delta {e}_{{{\rm{N}}}2{{\rm{d}}}\,{\mbox{-}}\,{{\rm{CC}}}}^{[J = 0]}({{\rm{6D}}}) - \delta {e}_{{{\rm{N}}}2{{\rm{d}}}\,{\mbox{-}}\,{{\rm{SAPT}}}}^{[J = 0]}({{\rm{6D}}})\right\vert \right. \\ 	 \left.+\left\vert \delta {e}_{{{\rm{N}}}2{{\rm{d}}}\,{\mbox{-}}\,{{\rm{CC}}}}^{[J = 0]}({{\rm{6D}}})\right\vert \right)/{e}_{{{\rm{N}}}2{{\rm{d}}}\,{\mbox{-}}\,{{\rm{CC}}}}^{[J = 0]}(4{{\rm{D}}}0),$$

obtained for the vibrational (*J* = 0) states (for the *α* values, see the “states.xls” file in an external repository^86^). Note that this ${\alpha }_{\max }$-based term is used to describe the energy dependence of the 6D – 4D deviations. Since the PES-related uncertainty is the most dominant contributor to the uncertainties of the computed energies, Eqs. (4)–(6) should provide realistic estimates for these uncertainties.

### The artificial localization model

To define artificially localized eigenstates for each of the four equivalent Z-shaped versions of the ^14^N_2_⋅^14^N_2_ dimer, the potential energy was drastically increased, to 0.5 *E*_h_, at those direct-product grid points which do not belong to the (*θ*_1_, *θ*_2_) coordinate range of the chosen version (see Fig. 6). This model, which artificially distinguishes the four versions of ^14^N_2_⋅^14^N_2_, requires the execution of four nuclear-motion computations. In this model, the wavefunction is excluded from three out of the four (*θ*_1_, *θ*_2_) coordinate quadrants. For each quadruply degenerate state *v* of this model, the artificially localized eigenfunctions form an orthonormal quadruplet $({\Lambda }_{v}^{{{\rm{I}}}},{\Lambda }_{v}^{{{\rm{II}}}},{\Lambda }_{v}^{{{\rm{III}}}},{\Lambda }_{v}^{{{\rm{IV}}}})$, where ${\Lambda }_{v}^{{{\mathcal{V}}}}$ is the localized eigenfunction of state *v* in version ${{\mathcal{V}}}$.

Using the set of eigenfunction quadruplets $({\Lambda }_{v}^{{{\rm{I}}}},{\Lambda }_{v}^{{{\rm{II}}}},{\Lambda }_{v}^{{{\rm{III}}}},{\Lambda }_{v}^{{{\rm{IV}}}})$, a rovibrational state of the ^14^N_2_⋅^14^N_2_ dimer can be expressed as

7 $$\psi \approx \mathop{\sum}_{v}{o}_{v}({s}_{v}^{{{\rm{I}}}}{\varLambda }_{v}^{{{\rm{I}}}}+{s}_{v}^{{{\rm{II}}}}{\varLambda }_{v}^{{{\rm{II}}}}+{s}_{v}^{{{\rm{III}}}}{\varLambda }_{v}^{{{\rm{III}}}}+{s}_{v}^{{{\rm{IV}}}}{\varLambda }_{v}^{{{\rm{IV}}}}),$$

where ${s}_{v}^{{{\mathcal{V}}}}=\pm 1$ is a sign associated with the $(\psi ,\,{\Lambda }_{v}^{{{\mathcal{V}}}})$ pair, and *o*_*v*_ stands for the unsigned wave function overlap between *ψ* and ${\Lambda }_{v}^{{{\mathcal{V}}}}$, which are the same for the four Z-shaped versions. Each wave function *ψ* can be labelled with (a) the four-fold degenerate state *v* having the largest *o*_*v*_ value, and (b) its sign quadruplet $({s}_{v}^{{{\rm{I}}}},{s}_{v}^{{{\rm{II}}}},{s}_{v}^{{{\rm{III}}}},{s}_{v}^{{{\rm{IV}}}})$. To avoid labeling inconsistencies, the (arbitrary) eigenfunction phases are synchronized by imposing the ${s}_{v}^{{{\rm{I}}}}=+1$ and ${\Lambda }_{v}^{{{\rm{I}}}}={{{\mathcal{P}}}}_{2}{\Lambda }_{v}^{{{\rm{II}}}}={{{\mathcal{P}}}}_{1}{\Lambda }_{v}^{{{\rm{III}}}}={{{\mathcal{P}}}}_{1}{{{\mathcal{P}}}}_{2}{\Lambda }_{v}^{{{\rm{IV}}}}$ conditions upon each *v*, where the ${{{\mathcal{P}}}}_{1/2}$ operation interchanges the two ^14^N nuclei within monomer 1/2.

## Supplementary information

Supplementary Information

## Data Availability

The computational data obtained during this study are available under the following OSF repository: 10.17605/OSF.IO/RJ6XB. This repository consists of two main units: (a) “pots.zip”, which contains FORTRAN implementations of the N2d-H0B, N2d-CC, and N2d-SAPT PESs, as well as (b) “rovib.zip”, a compressed archive of bound-state rovibrational energies for *J* ≤ 10, internal-coordinate wavefunction (quasi) densities, rigid-rotor decomposition files, and convergence tests using various basis functions and PESs.
