# Supplementary material for: Rovibrational dynamics of the quasistructural N2 dimer
Source: Commun Chem. 2025 Nov 7;8:339. doi: 10.1038/s42004-025-01716-7 (PMC12595043; doi:10.1038/s42004-025-01716-7)
Supplement: Supplementary file 1 — Supplementary Information [file 42004_2025_1716_MOESM1_ESM.pdf]

Supporting Information for the paper "Rovibrational dynamics of the quasistructural N_2 dimer" by Tóbiás *et al.*

Supplementary Note 1. Focal-point analysis for three distinguished stationary points of the N_2 dimer

Relying on the focal-point analysis (FPA) scheme,[1,2] the following approximation has been employed to determine the interaction energy at a specific $N_2{\cdot}N_2$ geometry:

$$\Delta E_{\rm int} \approx \Delta E_{\rm int}^{\rm HF} + \delta E_{\rm int}^{\rm CCSD} + \delta E_{\rm int}^{\rm (T)} + \delta E_{\rm int}^{\rm T} + \delta E_{\rm int}^{\rm (Q)} + \delta E_{\rm int}^{\rm Q} + \delta E_{\rm int}^{\rm (P)} + \delta E_{\rm int}^{\rm CV} + \delta E_{\rm int}^{\rm R}, \tag{S1}$$

whereby $\Delta E_{\rm int}^{M}$ and $\delta E_{\rm int}^{M}$ are the interaction energy and its increment, respectively, related to method M. In Eq. (S1), the following abbreviations are used: HF = Hartree–Fock, CCSD = coupled-cluster singles and doubles, (T)/T = perturbative/iterative triples, (Q)/Q = perturbative/iterative quadruples, (P) = perturbative pentuples, and CV/R = core-valence/relativistic effect. The $\delta E_{\rm int}^{M}$ increments of Eq. (S1) can be computed as

$$\begin{aligned}
\delta E_{\rm int}^{\rm CCSD} &= \Delta E_{\rm int}^{\rm CCSD} - \Delta E_{\rm int}^{\rm HF},\\
\delta E_{\rm int}^{\rm (T)} &= \Delta E_{\rm int}^{\rm CCSD(T)} - \Delta E_{\rm int}^{\rm CCSD},\\
\delta E_{\rm int}^{\rm T} &= \Delta E_{\rm int}^{\rm CCSDT} - \Delta E_{\rm int}^{\rm CCSD(T)},\\
\delta E_{\rm int}^{\rm (Q)} &= \Delta E_{\rm int}^{\rm CCSDT(Q)} - \Delta E_{\rm int}^{\rm CCSDT},\\
\delta E_{\rm int}^{\rm Q} &= \Delta E_{\rm int}^{\rm CCSDTQ} - \Delta E_{\rm int}^{\rm CCSDT(Q)},\\
\delta E_{\rm int}^{\rm (P)} &= \Delta E_{\rm int}^{\rm CCSDTQ(P)} - \Delta E_{\rm int}^{\rm CCSDTQ}\\
\delta E_{\rm int}^{\rm CV} &= \Delta E_{\rm int}^{\rm CCSD(T)[AE]} - \Delta E_{\rm int}^{\rm CCSD(T)},\\
\delta E_{\rm int}^{\rm R} &= \Delta E_{\rm int}^{\rm CCSD(T)[AE,\,DKH2]} - \Delta E_{\rm int}^{\rm CCSD(T)[AE]},
\end{aligned} \tag{S2}$$

where AE means all-electron correlations and DKH2 symbolizes the second-order Douglas–Kroll–Hess approach[3,4] (those coupled-cluster energies where "AE" is absent were derived in the frozen-core approximation). The CCSDT, CCSDT(Q), CCSDTQ, and CCSDTQ(P) single-point energies were obtained with the aid of the MRCC code,[5] while all the other electronic-structure computations were carried out with MOLPRO.[6]

As to the basis sets employed, the $\Delta E_{\rm int}^{\rm CCSD(T)[AE]}$ and $\Delta E_{\rm int}^{\rm CCSD(T)[AE,\,DKH2]}$ energies were determined with the aug-cc-pCVXZ[7] and aug-cc-pCVXZ-DK sets,[8] respectively, while the other terms of Eq. (S2) were evaluated with the aug-cc-pVXZ sets[9] and cardinal numbers $X \in \{2({\rm D}), 3({\rm T}), 4({\rm Q}), 5, 6\}$ (the only exception is the $\delta E_{\rm int}^{\rm (P)}$ increment, for which the cc-pVDZ basis set was utilized). In all interaction-energy computations related to FPA, counterpoise corrections[10] have been applied. Furthermore, when a $\Delta E_{\rm int}^{M}$ or a $\delta E_{\rm int}^{M}$ term is available for $X > 2$, this quantity was extrapolated to the CBS limit with the help of a $[X-1, X]$ (two-point) extrapolation schemes. For the HF interaction energy,

$$\Delta E_{\rm int}^{{\rm HF}[X,X+1]} = \Delta E_{\rm int}^{{\rm HF},X} + \frac{\Delta E_{\rm int}^{{\rm HF},X+1} - \Delta E_{\rm int}^{{\rm HF},X}}{1 - (1 + \frac{1}{X+1}) \exp\left\{9\left(\sqrt{X} - \sqrt{X+1}\right)\right\}} \tag{S3}$$

was used,[11] while the electron-correlation increments were derived through[12]

$$\delta E_{\rm int}^{{\rm corr}[X,X+1]} = \delta E_{\rm int}^{{\rm corr},X} + \frac{\delta E_{\rm int}^{{\rm corr},X+1} - \delta E_{\rm int}^{{\rm corr},X}}{1 - \left(\frac{X}{X+1}\right)^3}, \tag{S4}$$

where the cardinal numbers corresponding to the individual energy terms are placed in superscript.

For the FPA protocol, the geometries of the three selected stationary points, *i.e.*, the Z-, the T-, and the H-shaped forms, have been partially optimized. During these local searches, the r_1 and r_2 coordinates were kept fixed at their equilibrium values obtained at the frozen-core aug-cc-pV5Z CCSD(T) level, whereas the other coordinates (namely, R, θ_1, θ_2, and ϕ) were optimized at the frozen-core and counterpoise-corrected aug-cc-pV5Z CCSD(T)-F12b level[13] (for the optimized values of these four intermonomer coordinates, see Supplementary Table 2).

Supplementary Table 1: Focal-point analysis results for three selected stationary points of the N_2 dimer[a]

X(comb)	$\Delta E_{\rm int}^{\rm HF}$	$\{\delta E_{\rm int}^{\rm MP2}\}$	$\delta E_{\rm int}^{\rm CCSD}$	$\delta E_{\rm int}^{\rm (T)}$	$\delta E_{\rm int}^{\rm T}$	$\delta E_{\rm int}^{\rm (Q)}$	$\delta E_{\rm int}^{\rm Q}$	$\delta E_{\rm int}^{\rm (P)}$	$\delta E_{\rm int}^{\rm CV}$	$\delta E_{\rm int}^{\rm R}$	$\Delta E_{\rm int}$
					**Z-shaped structure (C_{2h})**						
					{6-31G*(0.25):		1.4}				
2	74.1	{−182.9}	−130.3	−24.3	2.7	−4.7	1.3		−0.2	0.1	−81.3
3	73.7	{−199.6}	−142.7	−28.5	3.5	−5.3			−0.2	0.1	−98.1
4	73.8	{−205.3}	−146.7	−29.8	3.7				−0.2	0.1	−103.3
5	73.5	{−207.7}	−148.2	−30.4					−0.2	0.1	−105.5
6	73.5	{−209.5}	−149.5								−106.8
[2, 3]	73.7	{−206.6}	−147.9	−30.3	3.8	−5.6			−0.2	0.1	−105.0
[3, 4]	73.8	{−209.5}	−149.6	−30.7	3.8				−0.2	0.1	−107.1
[4, 5]	73.4	{−210.2}	−149.8	−31.0					−0.2	0.1	−107.9
[5, 6]	73.5	{−212.0}	−151.3								−109.3
**CBS**	**73.5(1)**	**{−212(2)}**	**−151.3(15)**	**−31.0(3)**	**3.8(0)**	**−5.6(3)**	**1.3(1)**	**0.0(3)**	**−0.2(0)**	**0.1(0)**	**−109.3(26)**
					**T-shaped structure (C_{2v})**						
					{6-31G*(0.25):		1.1}				
2	64.2	{−164.7}	−119.0	−21.3	2.4	−4.2	1.1		−0.2	0.1	−76.9
3	62.1	{−179.7}	−130.3	−25.3	3.0	−4.7			−0.3	0.2	−94.1
4	62.0	{−184.7}	−133.9	−26.3	3.2				−0.3	0.2	−98.6
5	62.1	{−186.9}	−135.3	−26.8					−0.3	0.2	−100.4
6	62.0	{−188.3}	−136.3								−101.5
[2, 3]	61.9	{−186.0}	−135.1	−27.0	3.3	−5.1			−0.3	0.2	−100.9
[3, 4]	62.0	{−188.3}	−136.5	−27.0	3.3				−0.3	0.2	−102.2
[4, 5]	62.1	{−189.2}	−136.8	−27.3					−0.3	0.2	−102.7
[5, 6]	62.0	{−190.2}	−137.7								−103.7
**CBS**	**62.0(1)**	**{−190(1)}**	**−137.7(9)**	**−27.3(3)**	**3.3(0)**	**−5.1(3)**	**1.1(0)**	**0.0(3)**	**−0.3(0)**	**0.2(0)**	**−103.7(19)**
					**H-shaped structure (D_{2h})**						
					{6-31G*(0.25):		1.5}				
2	96.2	{−177.5}	−119.7	−22.2	2.5	−3.8	1.2	0.3	−0.2	−0.1	−46.1
3	96.9	{−200.6}	−137.1	−27.4	3.5	−4.7			−0.1	−0.1	−67.8
4	98.3	{−206.7}	−141.5	−28.8	3.8				0.0	−0.1	−71.7
5	98.1	{−209.9}	−143.6	−29.4					0.1	−0.1	−74.5
6	98.1	{−212.2}	−145.3								−76.2
[2, 3]	97.0	{−210.3}	−144.4	−29.6	3.9	−5.1			−0.1	−0.1	−77.3
[3, 4]	98.5	{−211.2}	−144.7	−29.8	4.1				0.1	−0.1	−75.9
[4, 5]	98.1	{−213.3}	−145.8	−30.0					0.2	−0.1	−77.5
[5, 6]	98.1	{−215.4}	−147.6								−79.3
**CBS**	**98.1(0)**	**{−215(2)}**	**−147.6(18)**	**−30.0(2)**	**4.1(2)**	**−5.1(4)**	**1.2(3)**	**0.3(1)**	**0.2(1)**	**−0.1(0)**	**−79.3(31)**

[a] The first column lists the X cardinal numbers and their $[X-1, X]$ combinations used to extrapolate the direct electronic-structure results to the complete basis set (CBS) limit. The other columns contain the interaction energies and their individual contributions, as specified in Eqs. (S1) and (S2). For the $[X-1, X]$ extrapolations, the formulas displayed in Eqs. (S3) and (S4) were applied. The boldfaced final estimates, reported in the rows starting with "CBS", correspond to the best available (in most cases extrapolated) predictions. The last(-two)-digit uncertainties of these best estimates are given in parentheses. The stability of the computationally demanding $\delta E_{\rm int}^{\rm Q}$ terms were checked by deriving their values with a smaller [6-31G*(0.25)] [14] basis set, but these auxiliary estimates, surrounded by braces, were not employed for extrapolation purposes. The even more expensive $\delta E_{\rm int}^{\rm (P)}$ correction could be computed only for the H-shaped structure; this value was added as an uncertainty to the $\delta E_{\rm int}^{\rm (P)}$ terms of the remaining two configurations.

Following the methodology outlined above, FPA analyses have been performed for the three selected stationary points of the $N_2 \cdot N_2$ dimer, with results displayed in Supplementary Table 1. In this table, the frozen-core second-order Møller–Plesset (MP2) increments, $\delta E_{\rm int}^{\rm MP2} = \Delta E_{\rm int}^{\rm MP2} - \Delta E_{\rm int}^{\rm HF}$, are also indicated in braces, but they were not taken into account in the final interaction energies because of their non-smooth convergence toward their complete basis set (CBS) limit. For the final (CBS) predictions of the specific energy contributions, conservative uncertainties have been guessed based on the level of their convergence. For a specific $\Delta E_{\rm int}$ interaction energy, its uncertainty is estimated with the sum of the uncertainties attached to its individual contributions. As apparent from Supplementary Table 2, the uncertainties due to the use of rigid monomers, ≈ 0.01 cm^{-1}, can be safely ignored.

Supplementary Table 2: Comparison of structural and interaction parameters at five salient $N_2{\cdot}N_2$ stationary points

Energy scheme[a]	Radial coordinates[b]			Angular coordinates[c]			Interaction parameters[d]		
	r_1	r_2	R	θ_1	θ_2	ϕ	ΔE_{intra}	$\delta E_{\text{int}}^{\text{FMC}}$	ΔE_{int}
**Z-shaped structure (global minimum, C_{2h})**									
N2d-H0B	1.101 400	1.101 400	3.995 4	50.13	50.13	0			–109.2
N2d-CC	1.098 656	1.098 656	4.010 6	49.61	49.61	0	0.000	0.000	–107.4
N2d-SAPT	1.088 831	1.088 831	4.048 9	49.75	49.75	0	0.000	0.000	–106.4
FPA	1.099 630	1.099 630	4.011 2	49.60	49.60	0			**–109.3(26)**
**T-shaped structure (first-order saddle point, C_{2v})**									
N2d-H0B	1.101 400	1.101 400	4.127 2	0	90	0			–104.0
N2d-CC	1.098 634	1.098 646	4.131 7	0	90	0	0.001	–0.001	–102.8
N2d-SAPT	1.088 810	1.088 820	4.146 8	0	90	0	0.000	0.000	–102.4
FPA	1.099 630	1.099 630	4.132 9	0	90	0			**–103.7(19)**
**X-shaped structure (second-order saddle point, D_{2d})**									
N2d-H0B	1.101 400	1.101 400	3.669 6	90	90	90			–91.6
N2d-CC	1.098 708	1.098 708	3.664 3	90	90	90	0.003	–0.002	–90.7
N2d-SAPT	1.088 893	1.088 893	3.666 5	90	90	90	0.005	–0.004	–92.4
**H-shaped structure (second-order saddle point, D_{2h})**									
N2d-H0B	1.101 400	1.101 400	3.703 8	90	90	0			–79.3
N2d-CC	1.098 732	1.098 732	3.697 7	90	90	0	0.003	–0.002	–78.0
N2d-SAPT	1.088 922	1.088 922	3.699 8	90	90	0	0.005	–0.004	–81.4
FPA	1.099 630	1.099 630	3.709 5	90	90	0			**–79.3(31)**
**I-shaped structure (fourth-order saddle point, $D_{\infty h}$)**									
N2d-H0B	1.101 400	1.101 400	4.942 9	0	0	0			–22.6
N2d-CC	1.098 715	1.098 715	4.960 2	0	0	0	0.006	–0.006	–21.7
N2d-SAPT	1.088 905	1.088 905	4.989 3	0	0	0	0.010	–0.009	–24.5

[a] Interaction-energy representations employed to compute the quantum-chemical parameters of this table (for details, see text).
[b] Radial coordinates of the dimer arrangements in Å (see Table 4 in the main text). r_1 and r_2 are constrained to the vibrationally averaged bond length of the isolated N_2 unit, 1.1014 Å, for the N2d-H0B potential, while the other energy schemes lead to (near-) equilibrium values for these two coordinates (cf. the equilibrium bond lengths given in Table 5 of the main text).
[c] Angular internal coordinates of the five dimer structures in degrees (see the Methods section in the main text).
[d] Interaction parameters, in cm^{-1}, for the individual dimer configurations. ΔE_{int} = interaction energy. ΔE_{intra} = intramonomer or deformation energy. $\delta E_{\text{int}}^{\text{FMC}}$ = flexible-monomer energy correction, defined as $\delta E_{\text{int}}^{\text{FMC}} = \Delta E_{\text{int}} - \Delta E_{\text{int}}^{\text{RMA}}$, which reflects the goodness of the rigid-monomer approximation (RMA).

Supplementary Note 2. Additional details about the PES construction procedure

For the intramonomer fit, 1720 distorted monomer configurations were generated using a uniform two-dimensional Sobol sequence[15,16] in the ± 0.15 Å range, around the approximate equilibrium bond length corresponding to the "CC"/"SAPT" level. Afterwards, the "CC"/"SAPT" intramonomer energies computed at these points were fitted to ascertain the $\tilde{r}_e$ bond length of the parametrized "CC"/"SAPT" deformation potential. To eliminate the relatively large (in fact, $6/115\ \text{cm}^{-1}$) errors characterizing the "CC"/"SAPT"-based stretch fundamental of the isolated N_2 molecule, the final intramonomer-energy function was fitted to deformation energies calculated from

$$\Delta E_{\text{intra}}^{\text{RKR}}(r_1, r_2) = V^{\text{RKR}}(r_1 + \delta r_e) + V^{\text{RKR}}(r_2 + \delta r_e) - 2V^{\text{RKR}}(r_e^{\text{RKR}}), \quad \text{(S5)}$$

where $V^{\text{RKR}}(r)$ is the ("experimental") Rydberg–Klein–Rees (RKR) potential of the $^{14}N_2$ species,[17,18] with an equilibrium bond length of $r_e^{\text{RKR}} = 1.097\,679(1)$ Å.[18] The shift $\delta r_e = r_e^{\text{RKR}} - \tilde{r}_e$ ensures that $\Delta E_{\text{intra}}^{\text{RKR}}(r_1, r_2)$ has its minimum at $(\tilde{r}_e, \tilde{r}_e)$, an important property when combining RKR with "CC"/"SAPT" in Eq. (3) of the main text.

For the N_2 dimer, ADS started with the computation of the lowest 3/150 states for the unsymmetrized 2D/4D vibrational Hamiltonian, drawing on the preliminary N2d-SAPT and N2d-CC PESs (see also Table 5 of the main text). In these 2D and 4D computations, 30×30 and $35\times15\times15\times14$ direct-product points were applied, respectively. From the 3/150 states, $3\times150 = 450$ approximate 6D eigenfunctions could be constructed by calculating the direct products of the 2D and 4D eigenvectors. These 450 states include 150 intermonomer vibrations below and somewhat

above the dissociation limit of the dimer, as well as their 150/150 combinations with the symmetric/antisymmetric intramonomer fundamentals. Within this 6D basis with $N = 30 \times 30 \times 35 \times 15 \times 15 \times 14$ points, the ith point was associated with the following (discrete) probability density:

$$P_i = \frac{1}{M} \sum_{j=1}^{M} c_{ij}^2, \tag{S6}$$

which corresponds to the average of the squared (approximate, "2D×4D"-like) wavefunction amplitudes determined for the $M = 150$ states. As long as the approximate eigenvectors of the M states are normalized, $\sum_{k=1}^{N} P_k = 1$ must hold, from which a cumulative probability density, $C_i = \sum_{k=1}^{i} P_k$, can be defined for the ith direct-product point.

Taking advantage of the C_i values, a direct-product point of index $l = \max\{1 \le i \le N : C_i \le u\}$ was selected as a new sample-point candidate, whereby $u \in [0, 1)$ is a random uniform variable [discrete inverse sampling; see, for instance, Eq. (34) of Ref. 19]. Out of these candidates, several points were discarded to meet the following criteria: (a) $F_l \ge 80/70/60/50/40/30/20/10/0\,\%$ must be satisfied for $20/30/40/50/60/70/80/90/100\,\%$ of the ADS sample, where $F_l = P_l / \max_{k=1}^{N} P_k$, and (b) the new points need to be sufficiently dissimilar from the already chosen sample points (the latter was monitored by the adaptive ϵ-metric approach[16,20] of the `autoPES` suite).

Of the four (asymptotic, main, hole-fixing, and minima-related) grid types, the ADS procedure was applied to replace only the main (short-range) grid generation process during the construction of the final N2d-CC/SAPT PESs (the other grid types were composed *via* the standard procedures[16,20] of the `autoPES` code). Since the non-ADS-based grid points do not belong to the 6D direct-product basis, an alternative "F-measure" is demanded to provide well-defined "statistical weights" for the intramonomer fit. If the kth point of the fitting dataset has a radial coordinate which is outside the lower/upper limits specified in Table 4 of the main text, then $\mathcal{F}^{[k]} = 0$, otherwise $\mathcal{F}^{[k]}$ is obtained *via* a linear interpolation from the $\{F_i : 1 \le i \le N\}$ factors. In this case, the kth fitting point ($1 \le k \le \mathcal{N}$) could be associated with a statistical weight $\mathcal{W}^{[k]} = \max(\mathcal{F}^{[k]}, \mathcal{F}^{\rm cut})$, where $\mathcal{N}$ is the number of fitting points in the actual intermonomer fit, and $\mathcal{F}^{\rm cut} = 0.001$ means a cut-off parameter adopted to retain a minimal effect of the fitted points with very small weights on the fit.

Supplementary Note 3. Additional details about the variational nuclear-motion computations

To compute the rovibrational states of the $N_2{\cdot}N_2$ dimer, the symmetry-adapted version[21] of the GENIUSH[22,23] code has been utilized, which solves the time-independent nuclear Schrödinger equation in a quasi-variational sense. Symmetry adaptation in GENIUSH requires that the points of the direct-product grid must be mapped onto each other under the effect of the symmetry operations, which is called here the grid-invariance criterion (GIC).

GIC is satisfied by the coordinate system defined in Table 4 of the main text, provided that (a) r_1 and r_2, as well as θ_1 and θ_2, are expressed with the same DVR (discrete variable representation)[24] grid, (b) θ_1 and θ_2 are sampled over the entire $[0, \pi)$ interval, and (c) ϕ is described with an even-point periodic DVR, like that advocated by Meyer[25] (this even-point formalism yields a number of ghost states, but their eigenvalues can be shifted above the highest eigenvalue of the Hamiltonian to inhibit their appearance among the desired states[25]). To check the fulfillment of GIC in a direct way for any coordinate system and MS group, a general subroutine has been added during this study to GENIUSH, which performs all the symmetry operations on the direct-product points in Cartesian space and matches the internal coordinates of the transformed Cartesian structures with the DVR grid points.

In the original implementation, symmetry adaptation was available in GENIUSH[21] only for $J = 0$. As part of the present study, the symmetry-adapted version of GENIUSH has been extended to treat the $J > 0$ case and compute rovibrational eigenstates by block diagonalization of the rovibrational Hamiltonian. The current implementation is limited to MS groups whose group elements involve equivalent rotations[26] by π radians around one of the molecule-fixed coordinate axes. Since this requirement is not met for the G_{16} group, the G_8 group was employed to construct group-theoretical projectors for the $J > 0$ case (note that violation of this requirement was not an issue in Refs. 27, 28, and 29 utilizing coupled nondirect-product basis sets). Thus, the computed $J > 0$ states could be automatically labelled by irreducible representations (irreps) of the G_8 group. Then, correlation rules between the G_8 and G_{16} irreps were determined, which helped convert the G_8 labels to their G_{16} counterparts. In those cases when two G_{16} irreps correlate with the same G_8 irrep, they were distinguished based on the characters of the $\mathcal{P}_{12}$ operation (*i.e.*, the one permuting the two monomers), which were calculated numerically from the GENIUSH eigenvectors.

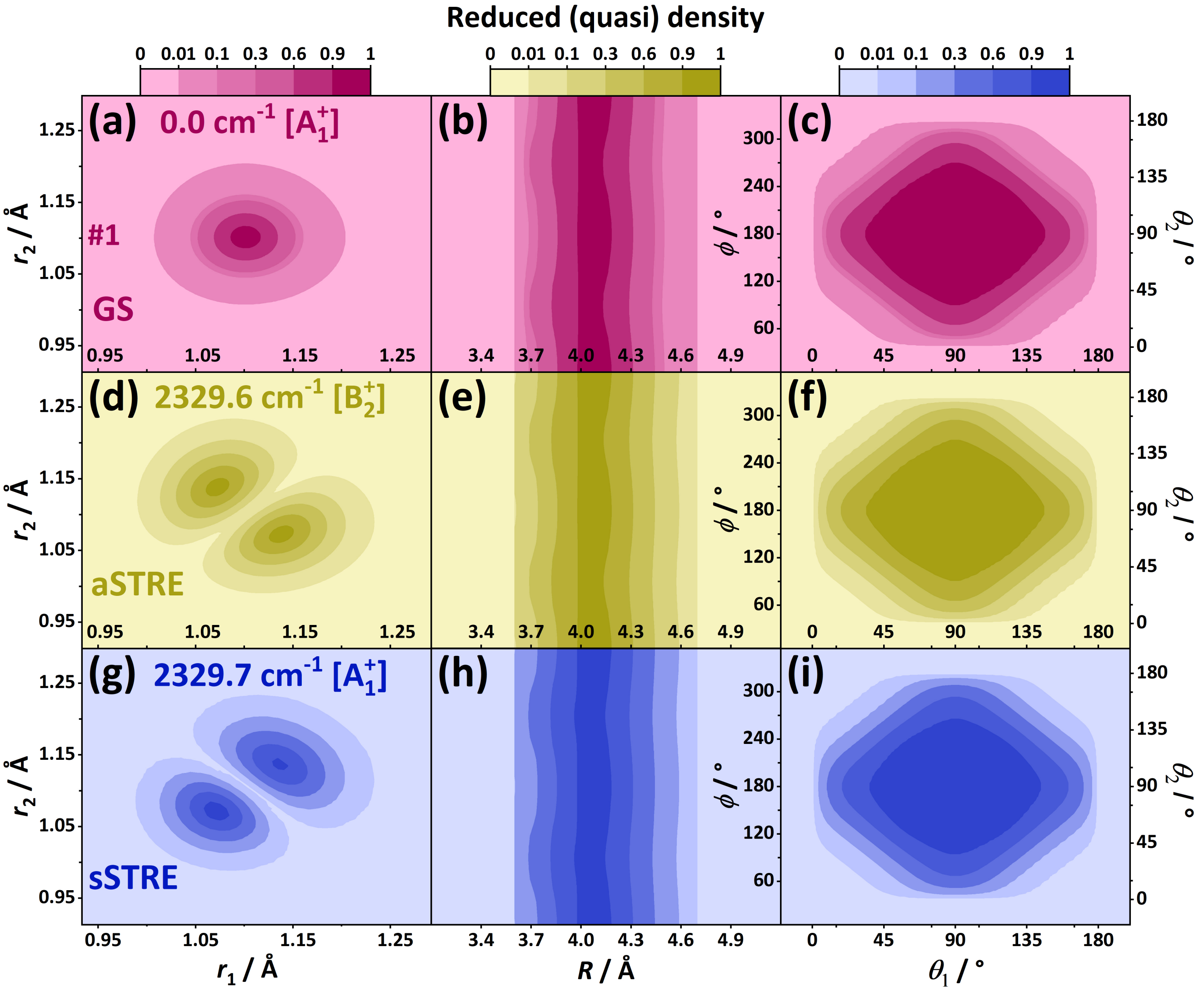

Supplementary Figure 1: Probability-density distributions calculated for the three vibrational states of $^{14}N_2{\cdot}^{14}N_2$ without intermonomer excitations. For the drawing conventions applied, see Fig. 5 in the main text.

Supplementary Note 4. Mass dependence of the first two excited vibrational energy

The first-order tetravariate Taylor polynomials for the lowest two excited vibrational energies of an arbitrary $N_2{\cdot}N_2$ isotopologue can be given as follows:

$$\varepsilon_1 \approx \varepsilon_0 + a'_{11}\Delta m'_1 + a''_{11}\Delta m''_1 + a'_{12}\Delta m'_2 + a''_{12}\Delta m''_2, \tag{S7}$$

$$\varepsilon_2 \approx \varepsilon_0 + a'_{21}\Delta m'_1 + a''_{21}\Delta m''_1 + a'_{22}\Delta m'_2 + a''_{22}\Delta m''_2, \tag{S8}$$

whereby (a) ε_0 is the lowest degenerate energy of the $^{14}N_2{\cdot}^{14}N_2$ species, (b) $\Delta m'_j = m'_j - m_0$ and $\Delta m''_j = m''_j - m_0$ indicate the two mass shifts within monomer j, (c) m'_j and m''_j denote the masses of the two atoms in monomer j, (d) m_0 symbolizes the mass of the ^{14}N isotope, while (e) a'_{ij} and a''_{ij} are the expansion coefficients representing the partial derivatives with respect to the four masses at $(m'_1, m''_1, m'_2, m''_2) = (m_0, m_0, m_0, m_0)$. Since the energy effect must be the same when interchanging $\Delta m'_i$ and $\Delta m''_i$, one obtains $a'_{ij} = a''_{ij} \equiv a_{ij}$, and then

$$\varepsilon_1 \approx \varepsilon_0 + a_{11}\Delta\mathcal{M}_1 + a_{12}\Delta\mathcal{M}_2, \tag{S9}$$

$$\varepsilon_2 \approx \varepsilon_0 + a_{21}\Delta\mathcal{M}_1 + a_{22}\Delta\mathcal{M}_2, \tag{S10}$$

where the monomer-mass shift $\Delta\mathcal{M}_j = \Delta m'_j + \Delta m''_j$ is introduced. In addition, swapping $\Delta\mathcal{M}_1$ and $\Delta\mathcal{M}_2$ may at most exchange ε_1 and ε_2, leading to

$$\varepsilon_2 \approx \varepsilon_0 + a_{21}\Delta\mathcal{M}_1 + a_{22}\Delta\mathcal{M}_2 = \varepsilon_0 + a_{11}\Delta\mathcal{M}_2 + a_{12}\Delta\mathcal{M}_1, \quad \text{(S11)}$$

from which $a_{11} = a_{22} \equiv a_1$ and $a_{12} = a_{21} \equiv a_2$ due to the arbitrariness of $\Delta\mathcal{M}_1$ and $\Delta\mathcal{M}_2$. Consequently,

$$\varepsilon_1 \approx \varepsilon_0 + a_1\Delta\mathcal{M}_1 + a_2\Delta\mathcal{M}_2, \quad \text{(S12)}$$

$$\varepsilon_2 \approx \varepsilon_0 + a_2\Delta\mathcal{M}_1 + a_1\Delta\mathcal{M}_2. \quad \text{(S13)}$$

These two expressions can be contracted for $i \in \{1, 2\}$ as follows:

$$\varepsilon_i \approx \varepsilon_0 + a_1\Delta\mathcal{M}_i + a_2\Delta\mathcal{M}_{3-i}, \quad \text{(S14)}$$

which is equivalent to Eq. (1) of the main text. However, to ensure the $\varepsilon_1 \leq \varepsilon_2$ relation, an appropriate convention must be applied for the indices of the monomers. This study follows an indexing convention where the $\Delta\mathcal{M}_1 \leq \Delta\mathcal{M}_2$ condition holds for the two monomers.

References

[1] Allen, W. D., East, A. L. L. & Császár, A. G. *Ab initio* anharmonic vibrational analyses of non-rigid molecules. In Laane, J., Dakkouri, M., van der Veken, B. & Oberhammer, H. (eds.) *Structures and conformations of nonrigid molecules*, 343–373 (Kluwer, Dordrecht, 1993).

[2] Császár, A. G., Allen, W. D. & Schaefer III, H. F. In pursuit of the *ab initio* limit for conformational energy prototypes. *J. Chem. Phys.* **108**, 9751–9764 (1998).

[3] Douglas, M. & Kroll, N. M. Quantum electrodynamical corrections to fine-structure of helium. *Ann. Phys.* **82**, 89–155 (1974).

[4] Hess, B. A. Relativistic electronic-structure calculations employing a 2-component no-pair formalism with external-field projection operators. *Phys. Rev. A* **33**, 3742–3748 (1986).

[5] MRCC website, last accessed on September 1, 2025.

[6] MOLPRO website, last accessed on September 1, 2025.

[7] Woon, D. E. & Dunning Jr., T. H. Gaussian basis sets for use in correlated molecular calculations. V. Core-valence basis sets for boron through neon. *J. Chem. Phys.* **103**, 4572–4585 (1995).

[8] De Jong, W. A., Harrison, R. J. & Dixon, D. A. Parallel Douglas–Kroll energy and gradients in NWChem: Estimating scalar relativistic effects using Douglas–Kroll contracted basis sets. *J. Chem. Phys.* **114**, 48–53 (2001).

[9] Kendall, R. A., Dunning Jr., T. H. & Harrison, R. J. Electron affinities of the 1st-row atoms revisited – Systematic basis sets and wave functions. *J. Chem. Phys.* **96**, 6796–6806 (1992).

[10] Boys, S. F. & Bernardi, F. The calculation of small molecular interactions by the differences of separate total energies. Some procedures with reduced errors. *Mol. Phys.* **19**, 553–566 (1970).

[11] Tasi, G. & Császár, A. G. Hartree–Fock-limit energies and structures with a few dozen distributed Gaussians. *Chem. Phys. Lett.* **438**, 139–143 (2007).

[12] Helgaker, T., Klopper, W., Koch, H. & Noga, J. Basis-set convergence of correlated calculations on water. *J. Chem. Phys.* **106**, 9639–9646 (1997).

[13] Adler, T. B., Knizia, G. & Werner, H.-J. A simple and efficient CCSD(T)-F12 approximation. *J. Chem. Phys.* **127** (2007).

[14] Hobza, P. & Šponer, J. Structure, energetics, and dynamics of the nucleic acid base pairs: nonempirical *ab initio* calculations. *Chem. Rev.* **99**, 3247–3276 (1999).

[15] Sobol', I. M. On the distribution of points in a cube and the approximate evaluation of integrals. *USSR Comput. Math. Math. Phys.* **7**, 784–802 (1967).

[16] Metz, M. P. & Szalewicz, K. Automatic generation of flexible-monomer intermolecular potential energy surfaces. *J. Chem. Theor. Comput.* **16**, 2317–2339 (2020).

[17] Bendtsen, J. The rotational and rotation-vibrational Raman spectra of $^{14}N_2$, $^{14}N^{15}N$ and $^{15}N_2$. *J. Raman. Spectrosc.* **2**, 133–145 (1974).

[18] Le Roy, R. J., Huang, Y. & Jary, C. An accurate analytic potential function for ground-state N_2 from a direct-potential-fit analysis of spectroscopic data. *J. Chem. Phys.* **125** (2006).

[19] Ismail, M. S. & Masseran, N. Risk assessment for extreme air pollution events using vine copula. *Stoch. Environ. Res. Risk Assess.* **38**, 2331—-2358 (2024).

[20] Metz, M. P., Piszczatowski, K. & Szalewicz, K. Automatic generation of intermolecular potential energy surfaces. *J. Chem. Theor. Comput.* **12**, 5895–5919 (2016).

[21] Fábri, C., Quack, M. & Császár, A. G. On the use of nonrigid-molecular symmetry in nuclear-motion computations employing a discrete variable representation: a case study of the bending energy levels of CH_5^+. *J. Chem. Phys.* **147**, 134101 (2017).

[22] Mátyus, E., Czakó, G. & Császár, A. G. Toward black-box-type full- and reduced-dimensional variational (ro)vibrational computations. *J. Chem. Phys.* **130**, 134112 (2009).

[23] Fábri, C., Mátyus, E. & Császár, A. G. Rotating full- and reduced-dimensional quantum chemical models of molecules. *J. Chem. Phys.* **134**, 074105 (2011).

[24] Harris, D. O., Engerholm, G. G. & Gwinn, W. D. Calculation of matrix elements for one-dimensional quantum-mechanical problems and the application to anharmonic oscillators. *J. Chem. Phys.* **43**, 1515–1517 (1965).

[25] Meyer, R. Trigonometric interpolation method for one-dimensional quantum-mechanical problems. *J. Chem. Phys.* **52**, 2053–2059 (1970).

[26] Bunker, P. R. & Jensen, P. *Molecular Symmetry and Spectroscopy* (NRC Research Press, Ottawa, 2006).

[27] Tennyson, J. & van der Avoird, A. Quantum dynamics of the van der Waals molecule $(N_2)_2$: An *ab initio* treatment. *J. Chem. Phys.* **77**, 5664–5681 (1982). Erratum: URL.

[28] Brocks, G. & van der Avoird, A. Infrared spectra of the van der Waals molecule $(N_2)_2$. *Mol. Phys.* **55**, 11–32 (1985).

[29] Aquilanti, V., Bartolomei, M., Cappelletti, D., Carmona-Novillo, E. & Pirani, F. The N_2–N_2 system: an experimental potential energy surface and calculated rotovibrational levels of the molecular nitrogen dimer. *J. Chem. Phys.* **117**, 615–627 (2002).
